# PAVE: Program for assembling and viewing ESTs

**DOI:** 10.1186/1471-2164-10-400

**Published:** 2009-08-26

**Authors:** Carol Soderlund, Eric Johnson, Matthew Bomhoff, Anne Descour

**Affiliations:** 1 BIO5 Institute, University of Arizona, Tucson, AZ 85721, USA

## Abstract

**Background:**

New sequencing technologies are rapidly emerging. Many laboratories are simultaneously working with the traditional Sanger ESTs and experimenting with ESTs generated by the 454 Life Science sequencers. Though Sanger ESTs have been used to generate contigs for many years, no program takes full advantage of the 5' and 3' mate-pair information, hence, many tentative transcripts are assembled into two separate contigs. The new 454 technology has the benefit of high-throughput expression profiling, but introduces time and space problems for assembling large contigs.

**Results:**

The PAVE (Program for Assembling and Viewing ESTs) assembler takes advantage of the 5' and 3' mate-pair information by requiring that the mate-pairs be assembled into the same contig and joined by n's if the two sub-contigs do not overlap. It handles the depth of 454 data sets by "burying" similar ESTs during assembly, which retains the expression level information while circumventing time and space problems. PAVE uses MegaBLAST for the clustering step and CAP3 for assembly, however it assembles incrementally to enforce the mate-pair constraint, bury ESTs, and reduce incorrect joins and splits. The PAVE data management system uses a MySQL database to store multiple libraries of ESTs along with their metadata; the management system allows multiple assemblies with variations on libraries and parameters. Analysis routines provide standard annotation for the contigs including a measure of differentially expressed genes across the libraries. A Java viewer program is provided for display and analysis of the results. Our results clearly show the benefit of using the PAVE assembler to explicitly use mate-pair information and bury ESTs for large contigs.

**Conclusion:**

The PAVE assembler provides a software package for assembling Sanger and/or 454 ESTs. The assembly software, data management software, Java viewer and user's guide are freely available.

## Background

ESTs have been prevalent in genomic research since the first large scale EST project in 1991 [1]. There are many EST projects that study the gene content of genome, tissue, or condition-specific transcripts (e.g. see Additional file 1: List of EST papers, section 4). In October 2005, 454 Life Sciences released the GS 20 pyrosequencer that generates over 100,000 reads per run with an average length of 110 bases [2-4]. In January 2007, they released the GS FLX that generates over 200,000 reads with length between 200–300. Table 1 shows the growth of the number of ESTs in GenBank in relation to their length. Many of the short sequences released after 2005 have been generated by the GS 20 or GS FLX 454 (there is no explicit field in GenBank stating the type). With the release of the Titanium 454 in October 2008, which produces reads of length 400 [4], we can expect to see the prevalence of 454 ESTs of length 400+ grow quickly.

**Table 1:** ESTs added to GenBank during specified years based on length

Year	≤ 200	201–300	301–650	>650	Total
2004	240 k	321 k	2731 k	1809 k	5103 k
2005	163 k	238 k	3869 k	3424 k	7695 k
2006^a^	749 k	448 k	3912 k	2911 k	8022 k
2007^b^	1323 k	404 k	3707 k	3753 k	8688 k
2008	1429 k	1060 k	3541 k	4316 k	10348 k

^a ^The GS 20 454 was released in October 2005 with read lengths less than 100.

^b ^The GS FLX 454 was released in January 2007 with read lengths between 200–300.

Besides the 454 sequencer, the following are also next-generation sequencers that generate high-throughput short reads: Illumina Genome Analyzer [5] developed by Solexa (Cambridge, UN), Applied Biosystems SOLiD Sequencing [6,7], and Helicos GSS Sequencing [8]. The 454 sequencer comes with the Newbler assembler, and there are multiple assembly packages tested for the Illumina system [9-15]. However, these are all tested on small genomes, chromosomes or BACs, which will have much shallower coverage compared to EST contigs.

Many current EST projects have generated 454 data and either used traditional EST assembly approaches (e.g. [16]) or aligned the ESTs to a related genome or assembled transcripts (e.g. [17]; see Additional file 1: List of EST papers, section 4.B). Laboratories are now transitioning between the traditional Sanger ESTs and new 454 ESTs. For example, our laboratory has a full-length cDNA project using Sanger 5' and 3' ESTs, and three other projects that have a mix of 454 and Sanger ESTs. For our Sanger projects, we developed a software package called PAVE (Program for Assembling and Viewing ESTs) that utilizes mate-pair information. With the release of the 454 sequencer, we extended PAVE to work for the increased depth of the 454 EST data sets.

The ESTs generated by Sanger versus the 454 sequencer differ in number and length. The current Sanger ESTs have an average length of around 650 good bases, but the number of ESTs that are sequenced is generally low. The cDNA sample must first be cloned into a vector (typically either plasmid- or phage-based) to produce a cDNA library and then individual clones are isolated from the library and sequenced, which results in a few thousand clones being sequenced. For example, most maize libraries in GenBank have between 1000 and 10,000 ESTs. The 454 GS FLX sequencer can generate over 200,000 good ESTs per project, but at an average length of only 250 trimmed bases. The new 454 GS FLX Titanium is capable of generating over a million reads of 400 bases with reduced error [4]. This technology currently does not produce identifiable cDNA mate-pairs.

Sanger sequencing can produce mate-pairs where it is known which ESTs are mates based on their name. If the clone is full length, the 5' end will start at the beginning of the transcript, otherwise it can start anywhere within the original mRNA sequence. It is now relatively inexpensive to generate both the 3' and 5' reads of a clone, as the library only needs to be prepped once. To date no software exists that takes full advantage of mate-pair information in order to produce better contigs. CAP3 [18] uses mate-pair information to build contigs but does not require that they be in the same contig. Phrap [19] uses mate-pair information to flag potential chimeric clones by inserting the chimeric mate into a contig. Clustering programs, such as STACK [20] and PaCE [21], will use mate-pair information to join clusters, but these may be broken into multiple contigs when assembled by CAP3 or Phrap. By contrast, PAVE requires mate-pairs to be in the same contig. It does not allow mate-pairs to be split across contigs, and if none of the ESTs in the 5' and 3' sub-contigs overlap, they are joined by n's. PAVE has been used to assemble multiple projects including 797,619 maize ESTs from GenBank [22].

The advent of 454 sequences presents new challenges to assembly. The increased depth of the 454 data sets can cause CAP3 and other assembly programs to run out of memory. Moreover, assembling large contigs (e.g. > 1000 ESTs) is time-consuming. To address both problems, ESTs contained in another may be removed, such as performed by PlantGDB [23,24]. PAVE removes ("buries") many of the ESTs that are contained in another ("parent" EST) during assembly; after assembly, the buried ESTs are placed in their parents' respective contigs in order to retain the expression level.

There are quite a few packages for the pipeline processing that EST data requires (see Additional file 1: List of EST papers, section 2). For example, EST2uni [25] is a pipeline that trims and cleans ESTS using external programs such as Lucy [26], assembles the ESTs with CAP3 or TGICL [27], and has annotation capabilities. We find that ESTs generated from different technologies and laboratories require different trimming and cleaning processes, so these functions are not part of the PAVE software package. However, PAVE includes a data management system in order to allow assembling many libraries together while retaining the information about each library. The PAVE system contains a Java program called jPAVE that allows easy verification and display of assemblies in the PAVE MySQL database. The system supports annotation by UniProt [28] match, GC content, ORFs, R statistic [29] and comparison of contigs. The assembly software, data management software, and jPAVE viewer are freely available along with a user's guide [30].

## Implementation

The PAVE system is written in Perl, the jPAVE assembly viewer is written in Java, and the data is stored in a MySQL database. The inputs to PAVE are standard FASTA formatted sequence and optional quality files, which have been trimmed and cleaned.

### PAVE assembly

**Figure 1:**
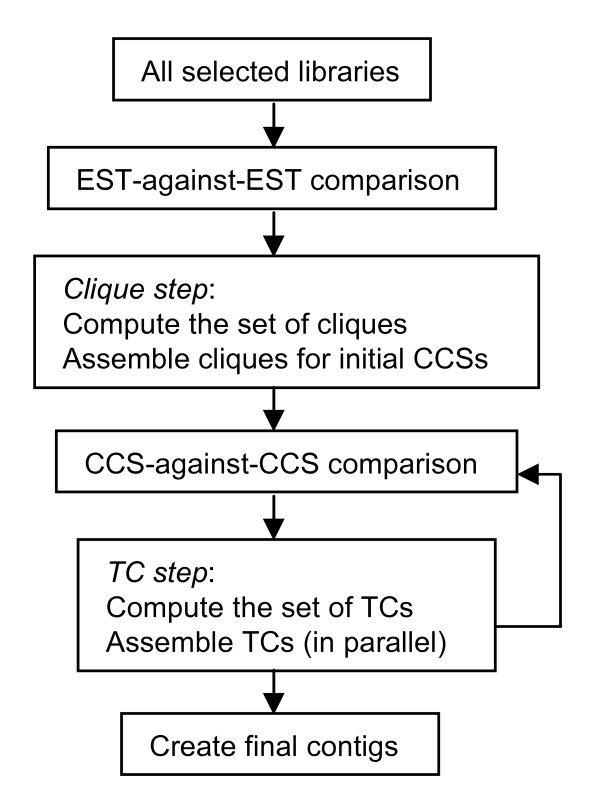
**A schema of the PAVE assembly algorithm**. The TC (transitive closure) loop is generally executed multiple times in order to merge contigs that have similar CCSs (contig consensus sequences). The user defines how many times the loop is executed, where for each loop a different set of parameters can be used. If the algorithm is being executed on a multi-processor machine, the user can request that the TC step use multiple processors.

As shown in Figure 1, the PAVE algorithm has a clique (fully connected graph) step, followed by one or more transitive closure (TC; connected graph) steps. The PAVE algorithm uses MegaBLAST [31] for similarity results and CAP3 [18] for assembly, with the following set of rules: (i) Mate-pairs must be in a contig together. If the mate-pairs assemble into two different contigs, the two sub-contigs are treated as a single contig. (ii) Contigs are incrementally built prioritized on bit scores and number of ESTs. Once a set of ESTs are in a contig together, they will never be split apart though they may be merged with others. (iii) CAP3 is only given sets of ESTs for assembly where the matched regions are correctly overlapping. Parameters governing these rules are provided by the user in a configuration file (see Additional file 2: Parameters and log files).

Guiding the system are the OK_OLAP and OK_CTG functions, which contain rules for acceptance or rejection of MegaBLAST and CAP3 output, respectively. OK_OLAP(*s*_*x*_, *s*_*y*_, *m*_*i*_, *d*_*i*_, *h*_*i*_) returns true if the sequences *s*_*x *_and *s*_*y *_have a minimum overlap of *m*, a minimum identity of *d%*, and a maximum overhang of *h*. The small amount of overhang allowed by *h *is necessary on the end of the overlapping portion to allow for poorly trimmed sequence. The parameters *m*_*i*_, *d*_*i *_and *h*_*i *_are set by the user in a configuration file (there are three or more sets as explained below). The OK_CTG(*rule*_*n*_) function analyzes the results of CAP3 to determine whether to accept or reject the contig. There are three sets of rules that have been designed by us based on inspection of contigs using the jPAVE graphical interface, and will be discussed in the section on heuristics.

The clique step creates seed contigs from the file of EST-against-EST comparison results, as follows: (i) Compute mate-pair cliques where each mate-pair must pass OK_OLAP(*s*_*x*_, *s*_*y*_, *m*_1_, *d*_1_, *h*_1_) with every other mate-pair in the clique. Each clique is assembled and if the CAP3 results pass OK_CTG(*rule1*), it is retained as a seed contig. (ii) Compute singleton cliques where each singleton must pass OK_OLAP(*s*_*x*_, *s*_*y*_, *m*_1_, *d*_1_, *h*_1_) with every other singleton in the clique. Each clique is assembled and if the CAP3 results pass the OK_CTG(*rule2*) function, it is retained as a seed contig. (iii) Each remaining mate-pair is made into a contig, where the mates are joined if they pass OK_OLAP(*s*_*x*_, *s*_*y*_, *m*_2_, *d*_2_, *h*_2_). (iv) Each remaining singleton is made into a contig. The purpose of assembling cliques is that the existence of a set of mate-pairs (or singletons) that all mutually overlap creates a strong contig and it reduces the time of the next step. This step produces the initial contig consensus sequences (CCS).

The TC step computes the set of TCs from the file of CCS-against-CCS comparison results. Each TC is a connected graph where each node is a contig, an edge exists between each two contigs that pass OK_OLAP(*s*_*x*_, *s*_*y*_, *m*_*i*_, *d*_*i*_, *h*_*i*_), and the edge is weighted by the bit-score. The nodes of a given TC are incrementally assembled together where the order is based on bit score and the number of ESTs. If a pair assembles correctly according to OK_CTG(*rule3*), the two nodes are collapsed into one. The TCs are assembled in parallel, i.e. each TC is assigned to a separate compute node for incremental assembly. The parallelism is implemented with the standard Unix fork function to take advantage of multiple processors.

The TC step is executed *n *times using the values (*m*_*i*_, *d*_*i*_, *h*_*i*_), *i *= 3 to *n*+3, where the *n *corresponding parameters are provided in the configuration file. For Sanger ESTs, we typically use two iterations, which merges over-split contigs; this approach of using a second assembly has been used by others, e.g. PlantGDB [23]. For 454 reads, we use up to eight iterations in order to allow for incremental burying, i.e. to prevent very large contigs from being assembled.

Two sub-contigs are not joined by n's until after assembly, at which point the orientation of the two sub-contigs needs to be computed. This is computed by a majority rule on the orientation and forward/reverse information of the ESTs.

### Heuristics

**Figure 2:**
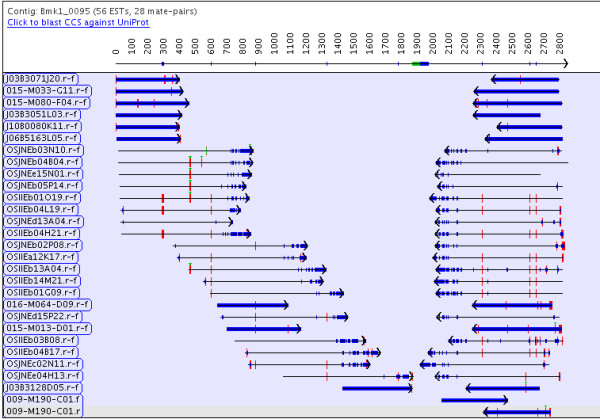
**A PAVE contig joined by n's**. No ESTs in the left and right sub-contig overlap, so the two sub-contigs are joined by 50 n's, which is indicated by the green box in the consensus sequence under the position 1900. The drawing of the EST indicates quality (blue is low quality), mismatches (red) and gaps (green). What appears to be thick blue lines are ESTs with no quality values, so all bases are low quality.

The OK_CTG function evaluates the output of CAP3 and accepts it if it results in one contig and rejects it if it results in more than two contigs (including singletons). If it results in two contigs, there must be more than one mate-pair bridging the two sub-contigs and all orientations must be consistent. These two rules are relaxed on the last iteration of assembling TCs in order to allow for the more common exceptions: (i) Two small sub-contigs may be bridged by one mate-pair. (ii) The sub-contigs may have mixed orientation, for example, both ends of the last mate-pair shown in Figure 2 assembled into the 3' contig. The heuristics try to determine if two-subcontigs are from the same transcript by using the counts of the 5', 3', un-complemented and complemented ESTs. These heuristics can never be 100 percent correct as the possibilities are endless, but are better than not joining the contigs or blindly joining them all (though there is an option to blindly join them).

A chimeric clone may have one half of the mate-pair belong in one contig and the other half in another contig. By forcing mate-pairs to be in contigs together, a chimeric clone will generally be in a contig of its own. The advantage is that it reduces chimeric multi-clone contigs resulting from chimeric clones. The disadvantage is that it may cause redundant contigs, as one half of the single mate-pair contig may align perfectly to another contig.

### Burying clones

To substantially decrease usage of computer time and space, ESTs are buried both before and during the assembly process, i.e. if EST B is contained in EST A, it can be "buried" in the "parent" EST A and removed from assembly. ESTs may be buried prior to assembly when one EST is contained in another based on the BLAST coordinates; the amount of mismatches allowed is a user-supplied parameter. During assembly, additional ESTs are buried if one is contained within another based on CAP3 coordinates, even though there may be mismatches between the buried and parent ESTs. For mate-pairs, both mates must be contained in the same parent clone in order to be buried. Buried ESTs are recorded in a file and when the PAVE algorithm completes and the contigs are loaded into the database, the buried clones are assigned the same contig as the parent.

The algorithm for burying ESTs (both initially and during assembly) ensures that the remaining unburied ESTs are sufficient to provide a good coverage of the contig. Briefly, the algorithm builds a containment tree and then performs a breadth-first traversal of the tree, retaining all top-level ESTs plus a number of ESTs from the next-highest levels based on configuration parameters. The breadth-first search serves to distribute the retained ESTs evenly across the contig. After retaining a sufficient number for good coverage, the rest of the ESTs in the tree are buried. For burial during assembly, the unburied ESTs are re-CAP'ed, and if they fail to assemble together, the attempt to bury the clones is rejected. Since this re-CAP takes time, the burial is only attempted if there are at least Y (default 100) ESTs in a contig and at least × (default 25) ESTs can be buried.

A post-processing routine reassembles the contigs that have buried ESTs using all ESTs in the contig, primarily in order to provide accurate coordinates for the buried clones. The re-CAP occasionally fails due to too many ESTs or due to the mismatches from the CAP3 buried ESTs; in these cases, the ESTs are positioned under their parent clone and shown in red.

### The PAVE system

As we have multiple PAVE projects, each with many EST libraries, it was advantageous to create an organized data management system as part of PAVE. This is done with a set of configuration files and a rigid directory structure, where there is a directory for libraries and another for assembly projects. By separating the libraries from the assemblies, different projects can be created with overlapping sets of libraries. The package provides four programs: (i) The loadLibrary.pl script loads one or more libraries of ESTs and metadata (i.e. organism, tissue, stage, etc) into the MySQL database. (ii) The runPAVE.pl script assembles the ESTs and enters the results into the database. (iii) The capBuried.pl script runs CAP3 on all the contigs with buried ESTs in order to assign correct coordinates. (iv) The jPAVE program provides some annotation and an interactive display.

After assembling the contigs, the runPAVE.pl script computes the R statistic [29], which identifies differentially expressed genes across the libraries. It also finds the longest clone (e.g. for microarrays) per contig, and marks contigs that are suspect based on having excessive mixed orientation or more than two stacks of ESTs. The jPAVE program's annotation module can be run to compute GC content, ORFs, SNPs, CCS against UniProt comparison, and CSS against CSS (to help detect paralogs and alternative splicing).

**Figure 3:**
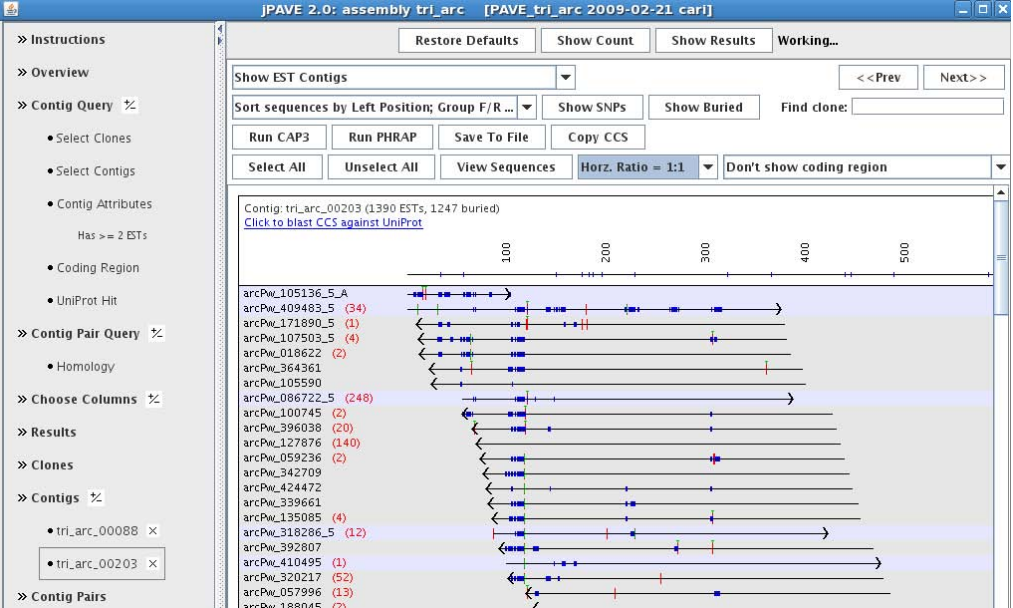
**The jPAVE interface**. Within the contig display, the numbers in parentheses are the number of ESTs buried in the corresponding EST. From the stand-alone version of jPAVE shown here, a set of ESTs can be selected, and CAP3 or Phrap can be executed on the ESTs. Also, ESTs from multiple contigs can be selected and assembled. The 'Contig Pairs' link lists all pairs of contigs that are similar; selecting a pair shows the nucleotide and amino acid alignment.

The jPAVE program can be run as either as a standalone program or a web applet. The initial window shows all PAVE projects, where any number can be selected for viewing (e.g. for comparing assemblies). As shown in Figure 3, jPAVE uses a BioMart [32] style query to allow easy complex queries on the annotation. Individual contigs can be displayed graphically or as base-pair sequences. By default, the buried ESTs are not displayed, which can save considerable time when displaying the contig; the number of buried ESTs is indicated next to each parent EST. The alignment of two CCSs can be viewed by nucleotide and amino acid similarity. In the standalone version, ESTs can be selected to assemble with CAP3 or Phrap using user-specified parameters.

## Results

Three sets of results are provided: (1) maize Sanger ESTs with mate-pairs, (2) trichome 454 ESTs, (3) a comparison of PAVE to CAP3 and TGICL with a benchmark set of Sanger mate-pair ESTs. The PAVE parameters used for each assembly are provided in Additional file 2. The Sanger and benchmark assemblies can be viewed from the PAVE site [30].

### Assembly of Sanger ESTs

The maize ESTs were downloaded from GenBank, then filtered to remove the ESTs less than 150 bases in length or submitted earlier than 1999. The resulting 797,619 ESTs had an average length of 585 bases and median of 605 bases. Unfortunately, there is no field in the GenBank record that explicitly identifies a 3' and 5' EST as mate-pairs, but the ESTs from our maize full length cDNA project [33] are named such that the mate-pairs are identified. All ESTs were entered into the PAVE database along with their library information, where there were 83 libraries with over 1000 clones (see [33]). Table 2 shows the results from assembling the ESTs. A significant metric is the 14,946 contigs joined by n's, where two contigs have been joined into one based on mate-pair information; most of these would have been reported as two contigs using other assembly programs. Likewise most of the 5,930 single mate-pair contigs would have been reported as singletons.

**Table 2:** PAVE assembly results

	Sanger maize	454 trichome
ESTs	797,619	415,559
Mate-pairs	127,452	n/a
Contigs^a^	51,202	24,437
Singletons	36,780	46,162
Contigs joined by n's	14,946	n/a
Mates joined by n's	5,930	n/a
Buried ESTs^b^	105,574	144,349
Time^c^	6.7 days	6 days

ESTs in contigs:		
= 2	13,465	9,926
3–5	13,526	8,249
6–10	8,493	2,860
11–20	6,514	1,463
21–50	6,125	1,049
51–100	2,090	445
101–1000	986	412
>1000	3	33

Length of contigs:		
1–100	0	4,769
101–500	22941	61,593
501–1000	33509	3,302
1001–2000	27569	867
2001–3000	3794	60
3001–4000	165	4
4001–5000	3	2
>5000	1	2

^a ^Does not include singletons.

^b ^No mismatches were allowed for the initial BLAST bury for the Sanger, whereas one mismatch was allowed for the 454.

^c ^The initial self-BLAST of the maize and trichomes ESTs took 33 CPU hours 9 CPU hours on a super-computer, respectively, which is not included in the time.

This maize Sanger assembly was executed on a system with two dual-core AMD Opteron 2 GHz processors and 12G RAM. Though the execution time is 6.7 days, very little human time is required to run the assembly. That is, the dataset does not have to be broken up, assembled separately and then merged, as would be necessary with programs that cannot handle datasets of this size. For example, the largest data set we can assemble on this machine with TGICL is 228,000 ESTs.

### Assembly of 454 ESTs

Our 454 data set is 415,559 ESTs from the stems of *Solanum arcanum *(Gang et al. manuscript in preparation) from the Solanum Trichome Project [34]. The average length of the cleaned ESTs is 218 bases and the median is 234 bases. The results are shown in Table 2. The largest contig is 19,739 ESTs with a 1283 bases consensus sequence; with the narrow depth, the ability to bury all but 342 of ESTs greatly speeds up assembly and display. In fact, it would not assemble without burying as CAP3 runs out of memory when assembling all 19,739 ESTs.

**Figure 4:**
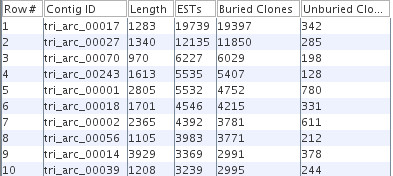
**10 largest 454 trichome contigs**. The jPAVE listing of the 454 contigs from the trichome assembly sorted on number of ESTs.

This trichome 454 assembly was executed on a system with four dual-core Intel Xeon 3.66 GHz processors and 14G RAM, which took almost as long as the much larger maize assembly, even though it was run on a faster machine. This is due to the number of deep contigs as illustrated in Figure 4, which shows the jPAVE listing of the top 10 contigs; in contrast, the largest maize contig is 2242 ESTs.

### Comparisons with other programs

We compared PAVE to TGICL and CAP3 for two reasons. First, the majority of EST papers use one of these two programs (see Additional file 1: List of EST papers, section 4). Second, all three assemblers use CAP3 for alignment, so that the comparison specifically addresses the effectiveness of using mate-pair information as a strict constraint, rather than variables in the alignment algorithm. To compare the assemblies, a set of benchmark contigs were created that contained 30,853 mate-pairs (61,706 ESTs) which aligned to 5437 of the KOME rice FL-cDNAs [35] (see Additional file 3: Benchmark contigs).

The benchmark ESTs were assembled with the three programs and the results were tested against the benchmark contigs to determine the number of split (i.e. false-negative) and merged (i.e. false-positive) contigs. A contig pair (*b*_1_, *b*_2_) was counted as merged if a pair of ESTs in an assembled contig were in different benchmark contigs *b*_1 _and *b*_2_. A contig pair (*a*_1_, *a*_2_) was counted as a split contig if a pair of ESTs in a benchmark contig were in different assembled contigs *a*_1 _and *a*_2_. Table 3 shows the numbers of split and merged contigs in addition to the number of split mate-pairs, singletons and contigs.

**Table 3:** Comparison of methods

	Contigs^a^	Singletons	Mate Splits	Contigs Split^a^	Contigs Merged	Time^b^
PAVE	5601^c^	0	0	186	0	3 h 25 m
TGICL	8888	71	16583	3401	12	25 m
CAP3	8811	342	16621	3454	15	2 h 12 m

^a ^Does not include singletons.

^b ^PAVE and TGICL used 4 processors, whereas CAP3 is not parallelized so used one processor.

^c ^3514 contigs are joined by n's where 53 of them are single-mate pair contigs.

As these are well-aligned ESTs, there are few merged contigs. Most of the split contigs for TGICL and CAP3 are due to mate-pairs that do not overlap, that is, 3514 PAVE contigs were joined by n's of which 53 are single mate-pairs. For example, Figure 2 shows two contigs joined by n's, which is a split contig in the CAP3 and TGICL assemblies. These split contigs account for many of the extra contigs in the TGICL and CAP3 assemblies, an excess which often causes the number of tentative genes to be over-estimated.

**Figure 5:**
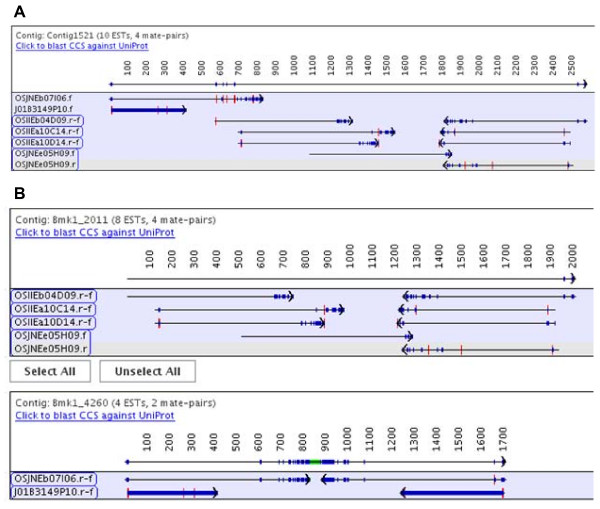
**An incorrect contig in CAP3 and TGICL**. This example shows where using mate-pairs prevents an incorrect join. (A) A contig found in both the CAP3 and TGICL assemblies where the first two ESTs are probably incorrectly joined as their 3' mates do not align. (B) The contig is split in PAVE, since the OSJNEb07I06 and J01B3149O10 mate-pairs must stay together and both the 5' and 3' align from these two clones.

As they all use CAP3 with identical parameters for alignment, the consensus sequences are very similar, with the average identity of each contig consensus sequence to the original FL-cDNA being over 99%. However, 4 TGICL consensus sequences and 5 CAP3 consensus sequences did not align well to their corresponding FL-cDNA due to bad joins which are avoided in PAVE by using mate-pair information; for example, see Figure 5.

## Conclusion

Since ESTs came into widespread use, the typical approach to assembling them has been to cluster the ESTs and assemble with CAP3 or Phrap. Most projects used CAP3 alone or TGICL for assembly, where TGICL clusters with a version of MegaBLAST and then assembles with CAP3 (see Additional file 1: List of EST papers, section 4). There are quite a few programs written specifically to cluster ESTs, but only miraEST [36] was specifically written to assemble ESTs (see Additional file 1: List of EST papers, section 1); note, CAP3 and Phrap were written for genome assembly. In comparison, there are many assemblers developed for whole genome sequencing based on Sanger reads, and a number of assemblers have emerged for Illumina reads.

As shown in our results, improved assemblies can be computed if both the 3' and 5' reads are used; this is similar to the improvements in assembly that are obtained when paired reads are used in whole genome assembly [37], and has also been shown for the Illumina paired short-reads [9]. In the early generation of the 3' and 5' Sanger ESTs, the mate-pair information could not be trusted, but for many years now, that has no longer been the case as laboratory tracking systems have improved. Though the use of Sanger sequencing for ESTs will probably decline, the historical data still has considerable value. As shown in Table 1 there are hundreds of thousands of Sanger ESTs available that should continue to be a valuable resource for years to come, and those with mate-pair information can take advantage of the PAVE assembler.

Though PAVE assembles the 454 ESTs with acceptable results, the new short read assemblers that are based on prefix trees and extensions (e.g. SSAKE [15]) or de Buijin graphs (e.g. Velvet [10], EULER-USR [9]) could provide better results when there is a lot of error or repeats. We are currently working on modifying PAVE so that it can use the results of various assemblies to take advantage of these custom assemblers and integrate the results with the PAVE mate-pair assemblies of Sanger reads.

The jPAVE assembly viewer provides an excellent way to evaluate contigs. We have assembled a large variety of libraries with different characteristics, and the occasional oddly-aligning contigs often cannot be explained nor anticipated. Having a versatile query and display program such as jPAVE is essential to understanding the wide range of problems that can occur with EST assembly. Moreover, it allows the biologist to diagnose the problems that occur with their specific libraries. For example, by assembling ESTs without the heuristics and then viewing them in jPAVE, the biologist can inspect the amount of chimerism and 3' slippage.

The PAVE system is created for large projects with multiple libraries. This allows comparing across libraries for differentially expressed genes, which will become more relevant with the large number of ESTs generated by the next-generation sequencing machines. The PAVE system also has a web interface (see [30] for examples), which allows extensive querying ability on the libraries (e.g. show the contigs that contain ESTs from one library that appear to be down-regulated in another). The web interface is not part of the distribution but is available upon request (email pave@agcol.arizona.edu). The software, jPAVE viewer and User's Guide are available from our PAVE website [30], where the User's Guide comes with a demonstration dataset.

## Availability and requirements

◦ Project name: PAVE

◦ Project home page: 

◦ Operating system: Unix/Linux

◦ Programming languages: Perl and Java

◦ Other requirements: Java 1.5 or higher, MySQL

◦ License: GNU Public License

◦ Any restriction to use by non-academics: none.

## Abbreviations

EST: Expressed Sequence Tags; GS: Genome Sequencer; BAC: Bacterial Artificial Chromosome; KOME: Knowledge-based Oryza Molecular biological Encyclopedia; ORF: Open Reading Frame; SNPs: Single Nucleotide Polymorphism; TC: Transitive Closure (i.e. connected graph)

## Authors' contributions

CS conceived the study and wrote the majority of the paper, with aid from all the other authors. EJ and MB provided most of the development work with aid from CS and AD. AD prepared the maize and trichome data sets.

## Supplementary Material

Additional file 1 **List of EST papers**. A partial list of EST papers published since 1999, with sections: 1. EST assembly software. 2. EST pre-processing, pipeline and viewing software. 3. EST annotation. 4. EST analysis for one for one or more libraries. 5. Related papers. 6. Typical references in EST papers. Click here for file

Additional file 2 **Parameters and log files**. The parameters and content the of log files for the maize, trichomes and benchmark assemblies. Click here for file

Additional file 3 **Benchmark contigs**. A description of the steps and parameters used to create the benchmark contigs. Click here for file
