# Supplementary material for: PAVE: Program for assembling and viewing ESTs
Source: BMC Genomics. 2009 Aug 26;10:400. doi: 10.1186/1471-2164-10-400 (PMC2748094; doi:10.1186/1471-2164-10-400)
Supplement: Additional file 1 — List of EST papers. A partial list of EST papers published since 1999, with sections: 1. EST assembly software. 2. EST pre-processing, pipeline and viewing software. 3. EST annotation. 4. EST analysis for one for one or more libraries. 5. Related papers. 6. Typical references in EST papers. [file 1471-2164-10-400-S1.doc]

# Additional file 1: EST papers

*This document provides a partial list of publications on ESTs, concentrating on papers published since 1999. Descriptions are given that are relevant to the PAVE paper.*

Contents

Additional file 1: EST papers [1](#__RefHeading___Toc112063102)

1. EST assembly software [1](#__RefHeading___Toc112063103)

A. EST assembly [1](#__RefHeading___Toc112063104)

B. Assembly of 2nd generation sequences [4](#__RefHeading___Toc112063105)

2. EST pre-processing, pipeline and viewing software [5](#__RefHeading___Toc112063106)

3. EST annotation [9](#__RefHeading___Toc112063107)

A. Polymorphisms (SNPs and Indels) [9](#__RefHeading___Toc112063108)

B. ORFs [10](#__RefHeading___Toc112063109)

4. EST analysis for one or more libraries [12](#__RefHeading___Toc112063110)

A. Sanger ESTs [12](#__RefHeading___Toc112063111)

B. Next-generation sequencing of ESTs [22](#__RefHeading___Toc112063112)

5. Related papers [25](#__RefHeading___Toc112063113)

A. Assorted [25](#__RefHeading___Toc112063114)

B. Alternative Splicing software [29](#__RefHeading___Toc112063115)

C. Full Length cDNA [31](#__RefHeading___Toc112063116)

6. Typical references in EST papers. [31](#__RefHeading___Toc112063117)

## 1. EST assembly software

*The following describes programs for assembling ESTs.*

### A. EST assembly

1. Bragg, L.M. and Stone, G. (2009) k-link EST Clustering: evaluating error introduced by chimeric sequences under different degrees of linkage. *Bioinformatics*.
2. Burke, J., D. Davison, and Hide. W. (1999) d2_cluster: a validated method for clustering EST and full-length cDNA sequences. *Genome Res* **9,** 1135-1142.

**d2_cluster** uses d2 (Wu et al. 1997, Biometrics 53:1431) for sequence similarity and transitive closure for the clusters. They present an evaluation of under and over clustering (in other words, type I and type II errors). The sensitivity and selectivity of d2_cluster are estimated to be >99.6% and 99.2%.

1. Chevreux, B., Pfisterer, T., Drescher, B., Driesel, A.J., Muller, W.E., Wetter, T. and Suhai, S. (2004) Using the miraEST assembler for reliable and automated mRNA transcript assembly and SNP detection in sequenced ESTs. *Genome Res*, **14**, 1147-1159.

**miraEST** uses SNP information to prevent the assembly of incorrect reads together. It iteratively computes HCRs (high confidence regions), performs automatic edits using the quality files and SNP detection, and extends the HCRs. There is an option to merge alleles after the 'pristine' transcripts are computed.

1. Christoffels, A., van Gelder, A., Greyling, G., Miller, R., Hide, T. and Hide, W. (2001) STACK: Sequence Tag Alignment and Consensus Knowledgebase. *Nucleic Acids Res*, **29**, 234-238.

**STACK** (often called STACKPACK) uses d2_cluster for clustering and Phrap for assembly.

1. Hazelhurst, S., Hide, W., Liptak, Z., Nogueira, R. and Starfield, R. (2008) An overview of the wcd EST clustering tool. *Bioinformatics*, **24**, 1542-1546.

**wcd** is a clustering algorithm that can be used with STACKPACK and can run in parallel.

1. Heber, S., Alekseyev, M., Sze, S.H., Tang, H. and Pevzner, P.A. (2002) Splicing graphs and EST assembly problem. *Bioinformatics*, 18 Suppl 1, S181-188.

They introduce the splicing graph that is a representation of all splicing variants. The graph is created with k-mers, and then successive vertices are collapsed if their in- and out-degree is one. Since a new edge will be created due to a sequencing error, error correction is performed by only accepting overlaps based on a set of constraints and using majority rules to determine a given base. A consensus base is computed for each position. The results were validated by viewing a few alternatively spliced genes (e.g. ADSL is about 20 kb long, contains 13 exons for an overall length of 2 kb).

1. Huang, X. and A. Madan. (1999) CAP3: A DNA sequence assembly program. *Genome Res* **9,** 868-877.

**CAP3** was developed for genomic sequence assembly but often used for ESTs.

1. Kalyanaraman, A., Aluru, S., Kothari, S. and Brendel, V. (2003) Efficient clustering of large EST data sets on parallel computers. *Nucleic Acids Res*, **31**, 2963-2974.

**PaCE** (Parallel clustering of ESTs) clusters ESTs with the aim that a cluster will represent a gene or paralogous genes. The algorithm first creates a generalized suffix tree, which is used to generate on-demand alignments (i.e. not all ESTs pairs need to be aligned) to form the clusters. Both steps are done in parallel. They use CAP3 to form the final contigs as that performed the best of three alignment programs. They tested their program by performing a spliced alignment of 168,200 ESTs to the Arabidopsis genome and using these as the benchmark clusters, which were compared to PaCE+CAP3 and CAP3 alone.

1. Lee, C., Grasso, C. and Sharlow, M.F. (2002) Multiple sequence alignment using partial order graphs. *Bioinformatics*, **18**, 452-464.

**POA** (Partial Order Alignment) is a graph representation of a multiple sequence alignment (MSA) that can itself be aligned directly by pair-wise dynamic programming. It accommodates sequencing errors, polymorphisms, and alternative splicing. It resulted in approximately 90,000 alignments from over 2 millions ESTs.

1. Malde, K., Coward, E. and Jonassen, I. (2003) Fast sequence clustering using a suffix array algorithm. *Bioinformatics*, **19**, 1221-1226.

An algorithm for clustering using suffix trees. The clusterings were compared to those produced by BLAST, d2_cluster and UIcluster.

1. Malde, K., Coward, E. and Jonassen, I. (2005) A graph based algorithm for generating EST consensus sequences. *Bioinformatics*, **21**, 1371-1375.

**xtract** is an algorithm that constructs a graph over sequence fragments of fixed size, and produces consensus sequences as traversals of this graph. They took the first 100 Unigene clusters, removed the mRNAs, and reclustered with xsact (Malde et al. 2003). The resulting clusters were assembled with xtract, Phrap, CAP3, and the TIGR assembler. They compared the results with the removed mRNAs. Xtract performed the best and CAP3 the second best.

1. Mudhireddy, R., Ercal, F. and Frank, R. (2004) Parallel hash-based EST clustering algorithm for gene sequencing. *DNA Cell Biol*, **23**, 615-623.

**HECT** (Hash based EST Clustering Tool) uses a hash-based algorithm for clustering where a parallel version has been tested on an IA-32 Linux cluster. For results, the number of clusters are compared with the number of Unigene clusters.

1. Parkinson, J., Guiliano, D.B. and Blaxter, M. (2002) Making sense of EST sequences by CLOBBing them. *BMC Bioinformatics*, **3**, 31.

**CLOBB** (Cluster on the basis of BLAST similarity) is a perl script that clusters BLAST output with a more intelligent algorithm than just transitive closure. It looks at where the overlap occurs and whether it is in a low quality region. It allows incremental additions to clusters. The paper compares the number of clusters formed with TIGR TCs and Unigenes. (www.nematodes.org/CLOBB)

1. Pertea, G., Huang, X., Liang, F., Antonescu, V., Sultana, R., Karamycheva, S., Lee, Y., White, J., Cheung, F., Parvizi, B. *et al.* (2003) TIGR Gene Indices clustering tools (TGICL): a software system for fast clustering of large EST datasets. *Bioinformatics*, **19**, 651-652.

**TGICL** uses a modified version of megaBLAST and CAP3. The system can run on multi-CPU architectures including SMP and PVM.

1. Picardi, E., Mignone, F. and Pesole, G. (2009) EasyCluster: a fast and efficient gene-oriented clustering tool for large-scale transcriptome data. *BMC Bioinformatics*, **10 Suppl 6**, S10.

Given ESTs and a sequenced genome, produces gene-oriented clusters.

1. Ptitsyn, A. and Hide, W. (2005) CLU: a new algorithm for EST clustering. *BMC Bioinformatics*, **6** Suppl 2, S3.

**CLU** is a match detection algorithm that ignores low-complexity regions like poly-tracts and short tandem repeats. It creates a hash tables then scores a sliding frame. The clustering merges each two sequences that score above a threshold, then the consensus sequence is used for subsequent matching. The clusters generated are compared with the d2_cluster results.

1. Trivedi N., Bischof J., Davis S., Pedretti K., Scheetz T.E., Braun T.A., Roberts C.A., Robinson N.L., Sheffield V.C., Soares M.B., and Casavant T.L. (2002) Parallel creation of non-redundant gene indices from partial mRNA transcripts. *Fut Generation Comput Syst* **18**, 863–870.

**UIcluster** uses a hash-based algorithm that has been parallelized using the MPI standard.

1. **Phrap** (www.phrap.org) was developed for genomic sequence assembly but often used for ESTs.

### B. Assembly of 2nd generation sequences

The following programs are all for BAC or whole genome assembly, but are listed here as they may (someday) work for ESTs.

1. Barker, M.S., Dlugosch, K.M., Reddy, A.C., Amyotte, S.N. and Rieseberg, L.H. (2009) SCARF: Maximizing next-generation EST assemblies for evolutionary and population genomic analyses. *Bioinformatics*.

**SCARF** assembles 454 ESTs against a high quality reference sequence.

1. Butler, J., MacCallum, I., Kleber, M., Shlyakhter, I.A., Belmonte, M.K., Lander, E.S., Nusbaum, C. and Jaffe, D.B. (2008) ALLPATHS: de novo assembly of whole-genome shotgun microreads. *Genome Res*, **18**, 810-820.

**ALLPATHS** was tested on 80x for 30-based reads up to 39Mb. They use a de Bruijin graph.

1. Chaisson, M.J., Brinza, D. and Pevzner, P.A. (2009) De novo fragment assembly with short mate-paired reads: Does the read length matter? *Genome Res*.

**EULER-USR** uses searches for a Eulerian path in a de Bruijn graph. Tested on E.coli and two BACs; with and without mate-pairs; up to 227x coverage.

1. Dohm, J.C., C. Lottaz, T. Borodina, and H. Himmelbauer. 2007. SHARCGS, a fast and highly accurate short-read assembly algorithm for de novo genomic sequencing. *Genome Res* **17:** 1697-1706.

**SHARCGS** uses a prefix tree with an extension algorithm. Tested on Illumina data from BACs, chromosomes and bacterial genomes.

1. Hernandez, D., Francois, P., Farinelli, L., Osteras, M. and Schrenzel, J. (2008) De novo bacterial genome sequencing: millions of very short reads assembled on a desktop computer. *Genome Res*, **18**, 802-809.

**EDENA** uses a classical overalp graph. Tested on 35-bp reads and 48x on two bacterium genomes.

1. Jeck, W.R., Reinhardt, J.A., Baltrus, D.A., Hickenbotham, M.T., Magrini, V., Mardis, E.R., Dangl, J.L. and Jones, C.D. (2007) Extending assembly of short DNA sequences to handle error. *Bioinformatics*, **23**, 2942-2944.

**VCAKE** uses k-mer extension.

1. Pop, M. and S.L. Salzberg. 2008. Bioinformatics challenges of new sequencing technology. *Trends Genet* **24:** 142-149.
2. Trombetti, G.A., R.J. Bonnal, E. Rizzi, G. De Bellis, and L. Milanesi. 2007. Data handling strategies for high throughput pyrosequencers. *BMC Bioinformatics* **8 Suppl 1:** S22.
3. Warren RL, Sutton GG, Jones SJ, Holt RA. 2007. Assembling millions of short DNA sequences using SSAKE. *Bioinformatics* 2007, **23**:500-501.

**SSAKE** uses a prefix tree and an extension algorithm. It was tested on metagenomic data from and small genomes.

1. Zerbino, D.R. and E. Birney. 2008. Velvet: algorithms for de novo short read assembly using de Bruijn graphs. *Genome Res* **18:** 821-829.

**Velvet** uses de Bruijn graphs and was tested on Solexa short reads (25-50 bp) and was tested on a prokaryote genome and a BAC.

## 2. EST pre-processing, pipeline and viewing software

*This section includes both downloadable software and web-based software for processing and analyzing ESTs.*

1. Adzhubei, A.A., Laerdahl, J.K. and Vlasova, A.V. (2006) **preAssemble**: a tool for automatic sequencer trace data processing. *BMC Bioinformatics*, **7**, 22.

Phred is run to base-call. Quality, vector, polyA and E.Coli contamination is screened with the Staden Pregap4 package. The results can be displayed on the web.

1. Ayoubi, P., Jin, X., Leite, S., Liu, X., Martajaja, J., Abduraham, A., Wan, Q., Yan, W., Misawa, E. and Prade, R.A. (2002) **PipeOnline** 2.0: automated EST processing and functional data sorting. *Nucleic Acids Res*, **30**, 4761-4769.

PipeOnline base-calls with phred, removes vector with crossmatch, assembles with Phrap, and BLASTall for annotation. The consensus sequences are compared with NCBI non-redundant protein databases. These records are matched with MPW-based functional directory (Selkov et al. NAR 26, 43) to add additional annotation. The database can be incrementally updated with new sequences and a new nr-database.

1. Baudet, C. and Dias, Z. (2006) Analysis of slipped sequences in EST projects. *Genet Mol Res*, **5**, 169-181.

They present three methods for detecting slipped sequences, i.e. when sequencing through a long polyA tail, there may be many signal peaks for each nucleotide which extends past the polyA, for example, the sequence 'actg' may end up being 'aaaaaccctttttgggggg'.

1. Close, T.J., Wanamaker, S., Roose, M.L. and Lyon, M. (2007) HarvEST: An EST Database and Viewing Software. *Methods Mol Biol*, **406**, 161-178.
2. D'Agostino, N., Aversano, M. and Chiusano, M.L. (2005) **ParPEST**: a pipeline for EST data analysis based on parallel computing. *BMC Bioinformatics*, 6 Suppl **4**, S9.

ParPEST uses PaCE for clustering and CAP3 for assembly. It uses RepeatMasker and the NCBI's VECTOR database for vector contamination, and RepeatMasker and RepBase for filtering and masking low complexity and interspersed repeats. The results are blasted against UniProt for annotation. The results are stored in a MySQL database with a web PHP-based interface. It is designed to run on a Beowulf cluster with Linux and the OSCAR 4.0 distributions for cluster management.

1. Forment, J., Gilabert, F., Robles, A., Conejero, V., Nuez, F. and Blanca, J.M. (2008) **EST2uni**: an open, parallel tool for automated EST analysis and database creation, with a data mining web interface and microarray expression data integration. *BMC Bioinformatics*, **9**, 5.

EST2uni pre-processes with Lucy, RepeatMasker and seqclean using NCBI's UniVec database. It assembles with CAP3 or TGICL. Unigene clusters can be computed of similar contigs. Annotation of SSRs with Sputnik (espressosoftware.com/pages/sputnik.jsp), SNPs are computed, in-silico PCR can be preformed, and GO, HMMER and orthologs can be computed. It has support for microarray expression integration. It uses a MySQL database and has web-based queries capabilities

1. Hotz-Wagenblatt, A., Hankeln, T., Ernst, P., Glatting, K.H., Schmidt, E.R. and Suhai, S. (2003) **ESTAnnotator**: A tool for high throughput EST annotation. *Nucleic Acids Res*, **31**, 3716-3719.

ESTAnnotator uses Phred for base-calling, Repeatmasker with a database of repetitive elements or UniVec for vector sequences, clustering was done by blasting against an organism specific database, CAP3 is used for the assembly and re-assembly of the consensus sequences. Annotation was performed with BLASTx against SWISSPROT and tBLASTx against ESTs from other organisms. A web-based graphical output displays the results.

1. Kumar, C.G., LeDuc, R., Gong, G., Roinishivili, L., Lewin, H.A. and Liu, L. (2004) **ESTIMA**, a tool for EST management in a multi-project environment. *BMC Bioinformatics*, **5**, 176.

ESTIMA (Expressed Sequence Tag Information Management and Annotation) consists of a SQL database schema, loading scripts and a web-based interface. The inputs are the chromatograms, EST sequence and quality files, EST contigs, and annotations. (titan.biotec.uiuc.edu/ESTIMA)

1. Latorre, M., Silva, H., Saba, J., Guziolowski, C., Vizoso, P., Martinez, V., Maldonado, J., Morales, A., Caroca, R., Cambiazo, V. *et al.* (2006) **JUICE**: a data management system that facilitates the analysis of large volumes of information in an EST project workflow. *BMC Bioinformatics*, **7**, 513.

A database management system that allows the user to upload sequences and compare the results of multiple assemblies.

1. Lee, B., Hong, T., Byun, S.J., Woo, T. and Choi, Y.J. (2007) **ESTpass**: a web-based server for processing and annotating expressed sequence tag (EST) sequences. *Nucleic Acids Res*.

ESTpass allows the user to submit up to 10000 ESTs to their website. It uses cross-match with a user supplied vector, adaptor or contaminants sequences to mask these sequences. Low-complexity regions are masked using RepeatMasker and a user-supplied repeat database. It detects chimeric ESTs that 'contain internally inserted contaminants' and removes them from further processing. D2_cluster and CAP3 are used for assembly. Chimerics are screened for in the resulting contigs by looking for barbell shaped contigs and blasting these against the nr database for confirmation. If found, they are excluded and the ESTs reassembled. The contigs are annotated by (1) BLASTx against the RefSeq protein database, (2) using the gene2go and gene2refseq files from Entrez gene, (3) BLAST against KEGG, (4) translate sequences in all 6 frame to search against InterProScan, and (5) TargetIdentifier to identify full-length transcripts.

1. Li, S. and H.H. Chou. 2004. **LUCY2**: an interactive DNA sequence quality trimming and vector removal tool. *Bioinformatics* **20:** 2865-2866.

Removes vector, poly-A and low quality from the ends.

1. Liang, C., Wang, G., Liu, L., Ji, G., Liu, Y., Chen, J., Webb, J.S., Reese, G. and Dean, J.F. (2007) **WebTraceMiner**: a web service for processing and mining EST sequence trace files. *Nucleic Acids Res*.

Trace files can be uploaded. Phred is run to base-call. The vector fragments, adapter/linker sequences, restriction sites, and polyA/polyT sites are identified and the results displayed.

1. Liang, C., Sun, F., Wang, H., Qu, J., Freeman, R.M., Jr., Pratt, L.H. and Cordonnier-Pratt, M.M. (2006) **MAGIC-SPP**: a database-driven DNA sequence processing package with associated management tools. *BMC Bioinformatics*, **7**, 115.

A data management package consisting of the database schema, loading scripts, program wrappers and query-based displays. The wrappers are for phred, cross-match and SSAHA.

1. Mao, C., Cushman, J.C., May, G.D. and Weller, J.W. (2003) **ESTAP**--an automated system for the analysis of EST data. *Bioinformatics*, **19**, 1720-1722.

ESTAP (EST Analysis Pipeline) cleans and trims the ESTs, flags chimeric, masks repeats, uses d2_cluster and CAP3, blasts against protein or DNA databases, and provides a user interface.

1. Masoudi-Nejad, A., Tonomura, K., Kawashima, S., Moriya, Y., Suzuki, M., Itoh, M., Kanehisa, M., Endo, T. and Goto, S. (2006) **EGassembler**: online bioinformatics service for large-scale processing, clustering and assembling ESTs and genomic DNA fragments. *Nucleic Acids Res*, **34**, W459-462.

A fasta sequence file can be uploaded to their website where it performs sequence cleaning, masking of repeats, vector and organelles, and assembles with CAP3. They created their own repeat and vector libraries.

1. Matukumalli, L.K., Grefenstette, J.J., Sonstegard, T.S. and Van Tassell, C.P. (2004) **EST-PAGE**--managing and analyzing EST data. *Bioinformatics*, **20**, 286-288.

EST-PAGE uses Phred for base-calling, cross-match for vector removal, assembly by CAP3, EST submission to Genbank, and a web interface. (EST-PAGE.binf.gmu.edu)

1. Muilu, J., Rodriguez-Tome, P. and Robinson, A. (2001) **GBuilder**--an application for the visualization and integration of EST cluster data. *Genome Res*, **11**, 179-184.

Gbuilder uses the AppLab server located at EBI for the following: CAP3 for assembly, CLEANUP (Grillo et al. 1996 CABIOS 12,1), and NCBI's DUST for masking low complexity regions. The tool has visualization capabilities to show similarities between sequences. Sequences may be edited. It can access different data sources and analysis applications on the internet using CORBA.

1. Nagaraj, S.H., Deshpande, N., Gasser, R.B. and Ranganathan, S. (2007) **ESTExplorer**: an expressed sequence tag (EST) assembly and annotation platform. *Nucleic Acids Res*.

ESTExplorer is a web-resource that uses SeqClean for vector removal using the NCBI UniVec database, polyA removal, trimming of low complexity and low quality sequence. It used RepeatMasker with Repbase to remove repeats. It uses CAP3 for assembly. For annotating the nucleotide sequence, it uses BLASTX against the NCBI non-redundant database, and BLAST2GO to map the result to GO terms. It uses ESTscan along with the 10 provide smat files (generated from mRNA sequences as training sets) to find the protein sequence, which is then run through InterPro and KOBAS.

1. Nagaraj, S.H., Gasser, R.B., Nisbet, A.J. and Ranganathan, S. (2008) In silico analysis of expressed sequence tags from Trichostrongylus vitrinus (Nematoda): comparison of the automated ESTExplorer workflow platform with conventional database searches. *BMC Bioinformatics*, **9 Suppl 1**, S10.
2. Nam, S.H., Kim, D.W., Jung, T.S., Choi, Y.S., Choi, H.S., Choi, S.H. and Park, H.S. (2009) PESTAS: a web server for EST analysis and sequence mining. *Bioinformatics*, **25**, 1846-1848.
3. Paquola, A.C., Nishyiama, M.Y., Jr., Reis, E.M., da Silva, A.M. and Verjovski-Almeida, S. (2003) **ESTWeb**: bioinformatics services for EST sequencing projects. *Bioinformatics*, **19**, 1587-1588.

A chromatogram is uploaded to their website, Phred is run for base-calling, cross-match is run to identify vector, adaptor sequence and the results are displayed.

1. Parkinson, J., Anthony, A., Wasmuth, J., Schmid, R., Hedley, A. and Blaxter, M. (2004) **PartiGene**--constructing partial genomes. *Bioinformatics*, **20**, 1398-1404.

PartiGene uses phred to base-call, cross_match to remove the vector, CLOBB for clustering, Phrap for assembly, blastx against a protein database, transeq (part of the EMBOSS package) to determine a tiling path from local regions of similarity, DECODER and ESTscan to obtain accurate peptide predictions, and find the longest ORG for the six frames if the previous two programs do not find an acceptable ORF.

1. Scheetz, T.E., Trivedi, N., Roberts, C.A., Kucaba, T., Berger, B., Robinson, N.L., Birkett, C.L., Gavin, A.J., O'Leary, B., Braun, T.A. *et al.* (2003) **ESTprep**: preprocessing cDNA sequence reads. *Bioinformatics*, **19**, 1318-1324.

Removes vector, poly-A and low quality from the ends.

1. Schmid, R. and Blaxter, M.L. (2008) **annot8r**: GO, EC and KEGG annotation of EST datasets. *BMC Bioinformatics*, **9**, 180.

Uses UniProt, GO, EC, KEGG and integrated with PartiGene.

1. Smith, R.P., Buchser, W.J., Lemmon, M.B., Pardinas, J.R., Bixby, J.L. and Lemmon, V.P. (2008) EST Express: PHP/MySQL based automated annotation of ESTs from expression libraries. *BMC Bioinformatics*, **9**, 186.

Phred, Crossmatch, BLASTN against UniGene, NCBI gene2unigene table, Entrez Gene, RefSEQ. PHP GUI and MySQL database.

1. Tang, Z., Choi, J.H., Hemmerich, C., Sarangi, A., Colbourne, J.K. and Dong, Q. (2009) ESTPiper--a web-based analysis pipeline for expressed sequence tags. *BMC Genomics*, **10**, 174.
2. Waegele, B., Schmidt, T., Mewes, H.W. and Ruepp, A. (2008) OREST: the online resource for EST analysis. *Nucleic Acids Res*, **36**, W140-144.

For mammalian or fungus using FunCat (Ruepp et al. NAR 32, 5539), GO and Morbid MAP (OMIM)

1. Zhu, T., Zhou, J., An, Y., Zhou, J., Li, H., Xu, G. and Ma, D. (2006) Construction and characterization of a rock-cluster-based EST analysis pipeline. *Comput Biol Chem*, **30**, 81-86.

This pipeline uses RepeatMasker with NCBI's VECTOR database, RepeatMasker and RepBase to mask repeats, PaCE for clustering, Phrap for assembly, mpiBLAST against protein databases, InterPro for domain and motif finding, and GoPipe.pl (Chen et al. 2005, Prog. Biochem. Biophys. 32, 187) to integrate BLAST and InterProScan results to obtain gene ontology annotation. This pipeline runs in parallel using the NPACI Rocks (www.rocksclusters.org) cluster software.

## 3. EST annotation

*This section only includes topics covered in the PAVE paper. Other types of annotation can be found in the Section V. Some of the pipelines in Section II also cover functional annotation.*

### A. Polymorphisms (SNPs and Indels)

1. Batley, J., G. Barker, H. O'Sullivan, K.J. Edwards, and D. Edwards. (2003) Mining for single nucleotide polymorphisms and insertions/deletions in maize expressed sequence tag data. *Plant Physiol* **132,** 84-91.

**AutoSNP** is a program they developed that runs d2cluster and CAP3 for assembly, then runs a program that computes SNPs. Contigs containing at least four reads were selected for SNP detection. The gap character (-) was treated as a base to permits the identification of insertion/deletion polymorphisms. When several SNPs were present in an alignment, a redundant co-segregation score was calculated for each SNP. In maize, they found one SNP per 600bp of aligned sequence for 5 read contigs and one per 100 bp for 20 read contigs. They report indels of size 1 to 26 bp with corresponding frequencies, where the first 8 are (1: 1014, 2:230, 3:168, 4:84, 5:48, 6:72, 7:25, 8:34, 9:6). Though indels generally occurred in non-coding regions, their data suggested that they are also found in coding regions.

1. Buetow, K.H., M.N. Edmonson, and A.B. Cassidy. (1999) Reliable identification of large numbers of candidate SNPs from public EST data. *Nat Genet* **21,** 323-325.

They use Phred and Phrap to base-call and build contigs, the dnadist and neighbour programs of PHYLIP, and a program they wrote to detects SNPs called DEMIGLACE. They filtered candidate SNPs based on the following filters: (i) any given slice where neighbouring sequence quality scores drop 40% or more; (ii) peak amplitude is below the fifteenth percentile of all base calls for that nucleotide type; (iii) sequence having a high number of disagreements with the consensus; (iv) any base call with an alternative call in which the peak takes up 25% or more of the area of the called peak; (v) exclude variations that occur in only one read direction. They calculated the posterior probability of a SNP using Baysian methods.

1. Garg, K., P. Green, and D.A. Nickerson. (1999) Identification of candidate coding region single nucleotide polymorphisms in 165 human genes using assembled expressed sequence tags. *Genome Res* **9,** 1087-1092.

They used Phred for base-calling and Phrap for assembly. Their SNP finding program used the following filters: (i) Two reads for each alternative base; (ii) Phred quality >20; (iii) average Phred quality 20 for the 5 bases on either side of the site; and (iv) an exact sequence match for the 5 bases on either side of suspected site. InDels were not considered. The SNP traces where manually inspected using Consed since there can be "systematic errores in the base-calling by Phred or alignments by Phrap*."* They determined the position of each candidate cSNP (coding SNP) in the codon, and whether the predicted change was synonymous (silent) or nonsynonymous (replacement). The identified a set of full length coding sequences from human genes that aligned to the contigs in order to find the coding SNPs. To minimize the chance of aligning a paralog CDS, alignments with high quality descrepancies (quality >=30) between the contig and the CDS exceeded 1% of the alignment were removed.

1. Huntley, D., A. Baldo, S. Johri, and M. Sergot. 2006**. SEAN**: SNP prediction and display program utilizing EST sequence clusters. *Bioinformatics* **22:** 495-496.

SEAN runs Phrap for assembly. To call a SNP, it requires that there be two occurrences of each base (e.g. at least 4 bases are necessary) and a window of 15 bases where there are no differences. A Java viewer is part of the package.

1. Marth, G.T., I. Korf, M.D. Yandell, R.T. Yeh, Z. Gu, H. Zakeri, N.O. Stitziel, L. Hillier, P.Y. Kwok, and W.R. Gish. 1999. A general approach to single-nucleotide polymorphism discovery. *Nat Genet* **23:** 452-456.

**PolyBayes** uses a Bayesian statistical model to determine if a site was polymorphic, which takes into account the depth of coverage, the base quality, and the a priori expected rate of polymorphic sites in the regions. They show results from SNP predications by aligning EST contigs to the human sequence. They found that paralogous sequences had a pair-wise dissimilarity rate higher than 0.02 compared to an average pair-wise polymorphism rate of 0.001, and used this to remove paralogous sequences from clusters.

1. Nelson, R.T., D. Grant, and R.C. Shoemaker. (2005) **ESTminer:** a suite of programs for gene and allele identification. *Bioinformatics* **21,** 691-693.

ESTminer is a collection of programs that analyzes ESTs from inbred genomes to identify unique genes within gene families. This paper discusses the problems with analyzing ESTs from crop species where many of them are polyploids. Hence, it is difficult to distinguish alleles of a single gene from similar paralogous genes. Their approach is to assemble (using CAP3) ESTs from an inbred genome to determine the consensus of gene families. Then BLAST is used to align ESTs to the consensus sequences to determine SNPs.

1. Picoult-Newberg, L., T.E. Ideker, M.G. Pohl, S.L. Taylor, M.A. Donaldson, D.A. Nickerson, and M. Boyce-Jacino. (1999) Mining SNPs from EST databases. *Genome Res* **9,** 167-174.

They assembled sequences with Phrap and called a SNP under the following conditions: (1) a perfect match of all bases in a window (5, 10 or 15) around the candidate SNP; (2) the candidate SNP cannot be an indel; (3) it cannot be within the first 100 bases of a sequence, (4) the mismatch must occur in more than one sequence.

1. Useche, F.J., G. Gao, M. Harafey, and A. Rafalski. (2001) High-throughput identification, database storage and analysis of SNPs in EST sequences. *Genome Inform Ser Workshop Genome Inform* **12,** 194-203.

The pipeline works with two different types of sequence assemblers, Phrap and CAT from DoubleTwist (www.doubletwist.com). It uses PolyBayes (Marth et al. 1999) for SNP and indel detection. They found in maize 1 SNP per 60-120 bp, whereas humans have 1 per 1000-1200bp. They also found that indels are high in maize.

### B. ORFs

*A mature cDNA (introns have been spliced out) has (i) a 5' UTR that often contains a stop code, (ii) the coding regions that starts with a start codon (ATG) and ends with a stop codon, and (iii) a 3' UTR. The start codon to the stop codon is referred to as the 'Open Reading Frame (ORF)' or the 'coding region'. Translation starts at the start codon, which is referred to as the 'initiation site', or the 'Translation Initiation Site (TIS)', where TIS is generally used in the context of uncovering typical features in the surrounding vicinity of the translation ATG, as the start codon is it not always the first ATG in the cDNA.*

1. Hatzigeorgiou, A.G., P. Fiziev, and M. Reczko. (2001) **DIANA-EST**: a statistical analysis. *Bioinformatics* **17,** 913-919.

This software used artificial neural networks to detect the start codon and coding region.

1. Iseli, C., C.V. Jongeneel, and P. Bucher. (1999) **ESTScan**: a program for detecting, evaluating, and reconstructing potential coding regions in EST sequences. *Proc Int Conf Intell Syst Mol Biol***,** 138-148.

ESTscan uses a Hidden Markov Model to locate the coding region. The algorithm attempts to correct for frame shifts and other types of sequencing errors. Since its transition probabilities are based on the codon usage biases of the species of interest, a large set of mRNA sequences with the boundaries of their coding regions annotated are required to train ESTscan.

1. Min, X.J., G. Butler, R. Storms, and A. Tsang. (2005) **OrfPredictor**: predicting protein-coding regions in EST-derived sequences. *Nucleic Acids Res* **33,** W677-680.

OrfPredictor is a web server designed for identifying protein-coding regions in EST-derived sequences. For query sequences with a BLASTX hit to a protein, the program predicts the coding regions based on the translation reading frames identified in BLASTX alignments, otherwise, it predicts the most probable coding region based on signals. It describes possibilities of combinations of 5'-UTR, stops before start, start, coding region, stop, poly-A, and provides 10 rules for finding the ORF.

1. Min, X.J., Butler, G., Storms, R. and Tsang, A. (2005) **TargetIdentifier**: a webserver for identifying full-length cDNAs from EST sequences. *Nucleic Acids Res*, **33**, W669-672.

TargetIdentifier is a web-based tool that determines if a cDNA is full-length, short full-length, possible-full-length, ambiguous, partial or 3' partial.

1. Nadershahi, A., S.C. Fahrenkrug, and L.B. Ellis. (2004) Comparison of computational methods for identifying translation initiation sites in EST data. *BMC Bioinformatics* **5,** 14.

Compared five methods to predict translation initiation sites in EST data: first-ATG, ESTScan, Diogenes, Netstart, and ATGpr.

1. Nishikawa, T., T. Ota, and T. Isogai (2000) Prediction whether a human cDNA sequence contains initiation codon by combining statistical information and similarity with protein sequences. *Bioinformatics* **16,** 960-967.

**ATGpr** (Salamov et al 1998, Bioinformatics, 14, 384), a program that predicts the initiation site using statistical information, was extended to use protein similarity matches. Their results show that prediction is > 80% if the sequence identity is > 40%, and that prediction is > 80% if the sequence identity is > 20% if used with the ATGpr score.

1. Schiex, T., J. Gouzy, A. Moisan, and Y. de Oliveira. (2003) **FrameD:** A flexible program for quality check and gene prediction in prokaryotic genomes and noisy matured eukaryotic sequences. *Nucleic Acids Res* **31,** 3738-3741.

FrameD was written to predict coding regions in prokaryotic but can be used for matured eukaryotic sequences (i.e. EST contigs). It can tolerate frameshifts as it uses DAG representation of all 6 reading frames and a learned set of k-mers (size 1 to 8). A set of training sets are available, or the user can create a new one. The program also uses protein similarity if available. FrameD can be run from the web or downloaded.

1. Wasmuth, J.D. and M.L. Blaxter (2004) **prot4EST**: translating expressed sequence tags from neglected genomes. *BMC Bioinformatics* **5,** 187.

prot4EST tries each of the following until one is successful: (1) BLAST against SwissProt, join and extend HSPs, (2) run ESTscan, (3) run DECODER, (4) identify the longest ORF from the six frame translations.

1. Zien, A., G. Ratsch, S. Mika, B. Scholkopf, T. Lengauer, and K.R. Muller (2000) Engineering support vector machine kernels that recognize translation initiation sites. *Bioinformatics* **16,** 799-807.

Their program uses a SVM to detect TISs using a window of 200 bases, so as to pick informative bases around the ATG.

## 4. EST analysis for one or more libraries

*These are papers that generated and analyzed a set of ESTs. For each paper, the assembly method is stated. A few paper have some additional detail about the analysis. Almost all papers BLASTed their contigs against one or more protein databases and assigned GO annotation with a program such as BLAST2go. Note:*

- *STACKPACK uses d2_cluster and CAP3*
- *AutoSNP uses d2_cluster and CAP3*
- *TGICL uses MegaBLAST and CAP3*
- *PAVE uses MegaBLAST and CAP3*
- *Paracel Transcript Assembly uses CAP3*

### A. Sanger ESTs

1. Abernathy, J.W., P. Xu, P. Li, D.H. Xu, H. Kucuktas, P. Klesius, C. Arias, and Z. Liu. (2007) Generation and analysis of expressed sequence tags from the ciliate protozoan parasite Ichthyophthirius multifiliis. *BMC Genomics* **8,** 176.

Vector NTI Advance 10 (Invitrogen, Carlsbad, CA)

1. Adzhubei, A.A., A.V. Vlasova, H. Hagen-Larsen, T.A. Ruden, J.K. Laerdahl, and B. Hoyheim. (2007) Annotated expressed sequence tags (ESTs) from pre-smolt Atlantic salmon (Salmo salar) in a searchable data resource. *BMC Genomics* **8,** 209.
2. Anderson, J.V., M. Delseny, M.A. Fregene, V. Jorge, C. Mba, C. Lopez, S. Restrepo, M. Soto, B. Piegu, V. Verdier, R. Cooke, J. Tohme, and D.P. Horvath. (2004) An EST resource for cassava and other species of Euphorbiaceae. *Plant Mol Biol* **56,** 527-539.

Analysis as done as in Anderson and Horvath (2001, Weeds Sci, 49, 581-589

1. Asamizu, E., Y. Nakamura, S. Sato, and S. Tabata. (2004) Characteristics of the Lotus japonicus gene repertoire deduced from large-scale expressed sequence tag (EST) analysis. *Plant Mol Biol* **54,** 405-414.

ESTs were clustered based on BLAST 98% identity for > 50 bases followed by Phrap.

1. Arunkumar, K.P., A. Tomar, T. Daimon, T. Shimada, and J. Nagaraju. 2008. WildSilkbase: an EST database of wild silkmoths. *BMC Genomics* **9:** 338.

Assembled with TGICL

1. Caprera, A., Lazzari, B., Stella, A., Merelli, I., Caetano, A.R. and Mariani, P. (2007) GoSh: a web-based database for goat and sheep EST sequences. *Bioinformatics*, **23**, 1043-1045.

The AutoSNP package.

1. Carre, W., X. Wang, T.E. Porter, Y. Nys, J. Tang, E. Bernberg, R. Morgan, J. Burnside, S.E. Aggrey, J. Simon, and L.A. Cogburn. 2006. Chicken genomics resource: sequencing and annotation of 35,407 ESTs from single and multiple tissue cDNA libraries and CAP3 assembly of a chicken gene index. *Physiol Genomics* **25:** 514-524.

Single-pass 5'-end sequencing assembled with CAP3.

1. Cerda, J., J. Mercade, J.J. Lozano, M. Manchado, A. Tingaud-Sequeira, A. Astola, C. Infante, S. Halm, J. Vinas, B. Castellana, E. Asensio, P. Canavate, G. Martinez-Rodriguez, F. Piferrer, J.V. Planas, F. Prat, M. Yufera, O. Durany, F. Subirada, E. Rosell, and T. Maes. 2008. Genomic resources for a commercial flatfish, the Senegalese sole (Solea senegalensis): EST sequencing, oligo microarray design, and development of the Soleamold bioinformatic platform. *BMC Genomics* **9:** 508.

Assembled with Phrap

1. Childs, K.L., Hamilton, J.P., Zhu, W., Ly, E., Cheung, F., Wu, H., Rabinowicz, P.D., Town, C.D., Buell, C.R. and Chan, A.P. (2007) The TIGR Plant Transcript Assemblies database. *Nucleic Acids Res*, **35**, D846-851.

Assembled with TGICL

1. Close, T.J., Wanamaker, S.I., Caldo, R.A., Turner, S.M., Ashlock, D.A., Dickerson, J.A., Wing, R.A., Muehlbauer, G.J., Kleinhofs, A. and Wise, R.P. (2004) A new resource for cereal genomics: 22K barley GeneChip comes of age. *Plant Physiol*, **134**, 960-968.

TGICL. They provide a discussion on what CAP3 parameters to change in order to change the number of contigs, singletons, paralogs and alleles. They also provide a nice description of preprocessing ESTs, including ones downloaded from Genbank.

1. D'Agostino, N., M. Aversano, L. Frusciante, and M.L. Chiusano. (2007) TomatEST database: in silico exploitation of EST data to explore expression patterns in tomato species. *Nucleic Acids Res* **35,** D901-905.

Uses ParPEST, which uses PaCE and CAP3 (see D'Agostino et al., section II).

1. Dong, Q., S.D. Schlueter, and V. Brendel. (2004) PlantGDB, plant genome database and analysis tools. *Nucleic Acids Res* **32,** D354-359.

Clustered with PaCE and aligned with CAP3.

1. Douglas, S.E., L.C. Knickle, J. Kimball, and M.E. Reith. (2007) Comprehensive EST analysis of Atlantic halibut (Hippoglossus hippoglossus), a commercially relevant aquaculture species. *BMC Genomics* **8,** 144.

Paracel Transcript Assembler 3.0 (Paracel Inc., Pasadena, CA), based on the CAP4 clustering algorithm

1. Dreyer, C., M. Hoffmann, C. Lanz, E.M. Willing, M. Riester, N. Warthmann, A. Sprecher, N. Tripathi, S.R. Henz, and D. Weigel. (2007) ESTs and EST-linked polymorphisms for genetic mapping and phylogenetic reconstruction in the guppy, Poecilia reticulata. *BMC Genomics* **8,** 269.

No assembly.

1. Ebbole, D.J., Y. Jin, M. Thon, H. Pan, E. Bhattarai, T. Thomas, and R. Dean. (2004) Gene discovery and gene expression in the rice blast fungus, Magnaporthe grisea: analysis of expressed sequence tags. *Mol Plant Microbe Interact* **17,** 1337-1347.

Assembled with Stackpack 2.1.

1. Ergen, N.Z. and Budak, H. (2009) Sequencing over 13 000 expressed sequence tags from six subtractive cDNA libraries of wild and modern wheats following slow drought stress. *Plant Cell Environ*, **32**, 220-236.

No assembly.

1. Fedorova, M., J. van de Mortel, P.A. Matsumoto, J. Cho, C.D. Town, K.A. VandenBosch, J.S. Gantt, and C.P. Vance. (2002) Genome-wide identification of nodule-specific transcripts in the model legume Medicago truncatula. *Plant Physiol* **130,** 519-537.

ESTs were compared against TIGR TCs

1. Fei, Z., X. Tang, R.M. Alba, J.A. White, C.M. Ronning, G.B. Martin, S.D. Tanksley, and J.J. Giovannoni. (2004) Comprehensive EST analysis of tomato and comparative genomics of fruit ripening. *Plant J* **40,** 47-59.

TIGR TCs

1. Fernandez, P., Paniego, N., Lew, S., Hopp, H.E. and Heinz, R.A. (2003) Differential representation of sunflower ESTs in enriched organ-specific cDNA libraries in a small scale sequencing project. *BMC Genomics*, **4**, 40.

Assembled with CAP3 with overlap cutoff identity of 95% and minimal overlap of 25.

1. Flinn, B., C. Rothwell, R. Griffiths, M. Lague, D. DeKoeyer, R. Sardana, P. Audy, C. Goyer, X.Q. Li, G. Wang-Pruski, and S. Regan. (2005) Potato expressed sequence tag generation and analysis using standard and unique cDNA libraries. *Plant Mol Biol* **59,** 407-433.

Assembled with Paracel Transcript Assembly

1. Forment, J., J. Gadea, L. Huerta, L. Abizanda, J. Agusti, S. Alamar, E. Alos, F. Andres, R. Arribas, J.P. Beltran, et al. (2005) Development of a citrus genome-wide EST collection and cDNA microarray as resources for genomic studies. *Plant Mol Biol* **57,** 375-391.

Assembled with Phrap with minmatch 50, minscore 100, trim_equal 28. Modified with Consed.

1. Frentiu, F.D., Adamski, M., McGraw, E.A., Blows, M.W. and Chenoweth, S.F. (2009) An expressed sequence tag (EST) library for Drosophila serrata, a model system for sexual selection and climatic adaptation studies. *BMC Genomics*, **10**, 40.

Assembled with TGICL

1. Gilchrist, M.J., A.M. Zorn, J. Voigt, J.C. Smith, N. Papalopulu, and E. Amaya. (2004_ Defining a large set of full-length clones from a Xenopus tropicalis EST project. *Dev Biol* **271**, 498-516.

Using BLAST results (ungapped mode with mismatch penalty of -1 and maiximum e-value of 1e-40), clusters were built using double linkage, and were aligned with their own alignment program. They designed their algorithm so that EST clusters contained alternative spliced transcripts, unedited or mis-edited RNAs, or significantly polymorphic sequences from different strains.

1. Gonzalez-Ibeas, D., J. Blanca, C. Roig, M. Gonzalez-To, B. Pico, V. Truniger, P. Gomez, W. Deleu, A. Cano-Delgado, P. Arus, F. Nuez, J. Garcia-Mas, P. Puigdomenech, and M.A. Aranda. (2007) MELOGEN: an EST database for melon functional genomics. *BMC Genomics* **8,** 306.

CAP or TGICL (via the EST2uni pipeline, bioinf.comav.upv.es/est2uni).

1. Hattori, J., T. Ouellet, and N.A. Tinker. (2005) Wheat EST sequence assembly facilitates comparison of gene contents among plant species and discovery of novel genes. *Genome* **48,** 197-206.

ESTs were clustered using transitive closure with output from BLAST at 1e-10, and assembled using Seqman II (DNASTAR Inc, Madison Wis).

1. Ho, C.L., Y.Y. Kwan, M.C. Choi, S.S. Tee, W.H. Ng, K.A. Lim, Y.P. Lee, S.E. Ooi, W.W. Lee, J.M. Tee, S.H. Tan, H. Kulaveerasingam, S.S. Alwee, and M.O. Abdullah. (2007) Analysis and functional annotation of expressed sequence tags (ESTs) from multiple tissues of oil palm (Elaeis guineensis Jacq.). *BMC Genomics* **8,** 381.

Assembled with CAP3

1. Hubbard, S.J., Grafham, D.V., Beattie, K.J., Overton, I.M., McLaren, S.R., Croning, M.D., Boardman, P.E., Bonfield, J.K., Burnside, J., Davies, R.M. *et al.* (2005) Transcriptome analysis for the chicken based on 19,626 finished cDNA sequences and 485,337 expressed sequence tags. *Genome Res*, **15**, 174-183.

Assembled with Phrap.

1. Jain, M., Shrager, J., Harris, E.H., Halbrook, R., Grossman, A.R., Hauser, C. and Vallon, O. (2007) EST assembly supported by a draft genome sequence: an analysis of the Chlamydomonas reinhardtii transcriptome. *Nucleic Acids Res*, **35**, 2074-2083.

Assembled their ESTs by aligning them to a draft genome - developed their own software to do this.

1. Jantasuriyarat, C., M. Gowda, K. Haller, J. Hatfield, G. Lu, E. Stahlberg, B. Zhou, H. Li, H. Kim, Y. Yu, R.A. Dean, R.A. Wing, C. Soderlund, and G.L. Wang. (2005) Large-scale identification of expressed sequence tags involved in rice and rice blast fungus interaction. *Plant Physiol* **138,** 105-115.

Assembled with early version of the PAVE algorithm.

1. Journet, E.P., D. van Tuinen, J. Gouzy, H. Crespeau, V. Carreau, M.J. Farmer, A. Niebel, T. Schiex, O. Jaillon, O. Chatagnier, L. Godiard, F. Micheli, D. Kahn, V. Gianinazzi-Pearson, and P. Gamas. 2002. Exploring root symbiotic programs in the model legume Medicago truncatula using EST analysis. *Nucleic Acids Res* **30:** 5579-5592.

Cluster with WU-BLAST and assembled with CAP3*.*

1. Jung, S., A. Abbott, C. Jesudurai, J. Tomkins, and D. Main. (2005) Frequency, type, distribution and annotation of simple sequence repeats in Rosaceae ESTs. *Funct Integr Genomics* **5,** 136-143.

Assembled using CAP3*.*

1. Jung, S., Staton, M., Lee, T., Blenda, A., Svancara, R., Abbott, A. and Main, D. (2008) GDR (Genome Database for Rosaceae): integrated web-database for Rosaceae genomics and genetics data. *Nucleic Acids Res*, **36**, D1034-1040.

Assembled with CAP3

1. Kang, L., X. Chen, Y. Zhou, B. Liu, W. Zheng, R. Li, J. Wang, and J. Yu. (2004) The analysis of large-scale gene expression correlated to the phase changes of the migratory locust. *Proc Natl Acad Sci U S A* **101,** 17611-17615.

Clustered with d2_cluster and assembled with Phrap.

1. Kuster, H., Becker, A., Firnhaber, C., Hohnjec, N., Manthey, K., Perlick, A.M., Bekel, T., Dondrup, M., Henckel, K., Goesmann, A. *et al.* (2007) Development of bioinformatic tools to support EST-sequencing, in silico- and microarray-based transcriptome profiling in mycorrhizal symbioses. *Phytochemistry*, **68**, 19-32.

Clustered with BLAST and assembled with CAP3.

1. Kim, H., C.J. Schmidt, K.S. Decker, and M.G. Emara. 2003. A double-screening method to identify reliable candidate non-synonymous SNPs from chicken EST data. *Anim Genet* **34:** 249-254.

Assembled with Phrap. Reading frame was found with ESTscan. SNPs were visualized with Consed.

1. Kunne, C., Lange, M., Funke, T., Miehe, H., Thiel, T., Grosse, I. and Scholz, U. (2005) CR-EST: a resource for crop ESTs. *Nucleic Acids Res*, **33**, D619-621.

Assembled with Stackpack.

1. Laffin, J.J., T.E. Scheetz, F. Bonaldo Mde, R.S. Reiter, S. Chang, M. Eyestone, H. Abdulkawy, B. Brown, C. Roberts, D. Tack, T. Kucaba, J.J. Lin, V.C. Sheffield, T.L. Casavant, and M.B. Soares. (2004) A comprehensive nonredundant expressed sequence tag collection for the developing Rattus norvegicus heart. *Physiol Genomics* **17,** 245-252.

UIcluster (see Trivedi et al. 2002, section I) groups ESTs into clusters.

1. Lanier, W., Moustafa, A., Bhattacharya, D. and Comeron, J.M. (2008) EST analysis of Ostreococcus lucimarinus, the most compact eukaryotic genome, shows an excess of introns in highly expressed genes. *PLoS ONE*, **3**, e2171.

Assembled with TGICL

1. Lazo, G.R., S. Chao, D.D. Hummel, H. Edwards, C.C. Crossman, N. Lui, D.E. Matthews, V.L. Carollo, D.L. Hane, F.M. You, et al. (2004) Development of an expressed sequence tag (EST) resource for wheat (Triticum aestivum L.): EST generation, unigene analysis, probe selection and bioinformatics for a 16,000-locus bin-delineated map. *Genetics* **168,** 585-593.

Assembled with Phrap, where parameters were set to allow like-sequences with 90% identity over a 100-base length to form contig clusters

1. Lazzari, B., A. Caprera, A. Vecchietti, A. Stella, L. Milanesi, and C. Pozzi. (2005) ESTree db: a tool for peach functional genomics. *BMC Bioinformatics* **6 Suppl 4,** S16.

AutoSNP for assembly and SNP finding.

1. Lazzari, B., Caprera, A., Cosentino, C., Stella, A., Milanesi, L. and Viotti, A. (2007) ESTuber db: an online database for Tuber borchii EST sequences. *BMC Bioinformatics*, **8 Suppl 1**, S13.

Assembled with CAP3, TrimEST (EMBOSS), Tandem Repeat Finder program [Benson, NAR 1999, 27:573], FrameFinder (bioweb.pasteur.fr/docs/man/man/ESTate.1.html)

1. Lee, Y., J. Tsai, S. Sunkara, S. Karamycheva, G. Pertea, R. Sultana, V. Antonescu, A. Chan, F. Cheung, and J. Quackenbush. (2005) The TIGR Gene Indices: clustering and assembling EST and known genes and integration with eukaryotic genomes. *Nucleic Acids Res* **33,** D71-74.

Assembled with TGICL.

1. Lee, S.H., Park, E.W., Cho, Y.M., Lee, J.W., Kim, H.Y., Lee, J.H., Oh, S.J., Cheong, I.C. and Yoon, D.H. (2006) Confirming single nucleotide polymorphisms from expressed sequence tag datasets derived from three cattle cDNA libraries. *J Biochem Mol Biol*, **39**, 183-188.

Assembled with Phrap. Polyphred for SNPs.

1. Li, L., J. Crabtree, S. Fischer, D. Pinney, C.J. Stoeckert, Jr., L.D. Sibley, and D.S. Roos. (2004) ApiEST-DB: analyzing clustered EST data of the apicomplexan parasites. *Nucleic Acids Res* **32,** D326-328.

Assembled using CAP3.

1. Lin, C., Mueller, L.A., Mc Carthy, J., Crouzillat, D., Petiard, V. and Tanksley, S.D. (2005) Coffee and tomato share common gene repertoires as revealed by deep sequencing of seed and cherry transcripts. *Theor Appl Genet*, **112**, 114-130.

Clustering using a 'Smith-Waterman type algorithm with word matching' and CAP3 using parameters -e 5000 -p 90 -d 10000 -b 60. ESTscan for coding frames. To detect paralog and allelic consensus sequences, they were self-BLASTed and those with 70% homology were plotted.

1. Liang, C., G. Wang, L. Liu, G. Ji, L. Fang, Y. Liu, K. Carter, J.S. Webb, and J.F. Dean. (2007) ConiferEST: an integrated bioinformatics system for data reprocessing and mining of conifer expressed sequence tags (ESTs). *BMC Genomics* **8,** 134.

WebTraceMiner for processing. No apparent assembly.

1. Lokko, Y., Anderson, J.V., Rudd, S., Raji, A., Horvath, D., Mikel, M.A., Kim, R., Liu, L., Hernandez, A., Dixon, A.G. *et al.* (2007) Characterization of an 18,166 EST dataset for cassava (Manihot esculenta Crantz) enriched for drought-responsive genes. *Plant Cell Rep*.

ESTs were clustered using a hash-based algorithm (Bixomax Informations, Martinsried, Germany) and assembled with CAP3.

1. Lu, C., J.G. Wallis, and J. Browse. (2007) An analysis of expressed sequence tags of developing castor endosperm using a full-length cDNA library. *BMC Plant Biol* **7,** 42.

Assembled with StackPack

1. Ma, H.M., S. Schulze, S. Lee, M. Yang, E. Mirkov, J. Irvine, P. Moore, and A. Paterson. (2004) An EST survey of the sugarcane transcriptome. *Theor Appl Genet* **108,** 851-863.

Sequences containing at least 100 continuous nucleotides with a phred score greater than 16 were clustered by Phrap with a minscore of 80. Assembled contigs were viewed/edited using Consed.

1. Maheswari, U., A. Montsant, J. Goll, S. Krishnasamy, K.R. Rajyashri, V.M. Patell, and C. Bowler. (2005) The Diatom EST Database. *Nucleic Acids Res* **33,** D344-347.

Assembled with CAP3.

1. Miller, R.T., A.G. Christoffels, C. Gopalakrishnan, J. Burke, A.A. Ptitsyn, T.R. Broveak, and W.A. Hide. (1999) A comprehensive approach to clustering of expressed human gene sequence: the sequence tag alignment and consensus knowledge base. *Genome Res* **9,** 1143-1155.

Uses d2_cluster for clustering and Phrap for assembly.

1. Mishra, R.N., P.S. Reddy, S. Nair, G. Markandeya, A.R. Reddy, S.K. Sopory, and M.K. Reddy. 2007. Isolation and characterization of expressed sequence tags (ESTs) from subtracted cDNA libraries of Pennisetum glaucum seedlings. *Plant Mol Biol* **64:** 713-732.

Assembled with Phrap

1. Mita, K., Morimyo, M., Okano, K., Koike, Y., Nohata, J., Kawasaki, H., Kadono-Okuda, K., Yamamoto, K., Suzuki, M.G., Shimada, T. *et al.* (2003) The construction of an EST database for Bombyx mori and its application. *Proc Natl Acad Sci U S A*, **100**, 14121-14126.

Uses BLAST and CLUSTAL

1. Moyle, R.L., M.L. Crowe, J. Ripi-Koia, D.J. Fairbairn, and J.R. Botella. 2005. PineappleDB: an online pineapple bioinformatics resource. *BMC Plant Biol* **5:** 21.

Assembled with Seqman (DNASTAR).

1. Nagel, J., Culley, L.K., Lu, Y., Liu, E., Matthews, P.D., Stevens, J.F. and Page, J.E. (2008) EST analysis of hop glandular trichomes identifies an O-methyltransferase that catalyzes the biosynthesis of xanthohumol. *Plant Cell*, **20**, 186-200.

Assembled with TGICL

1. Parkinson, J., C. Whitton, R. Schmid, M. Thomson, and M. Blaxter. (2004) NEMBASE: a resource for parasitic nematode ESTs. *Nucleic Acids Res* **32,** D427-430.

Clustered with BLAST and assembled with Phrap.

1. Paschall, J.E., M.F. Oleksiak, J.D. VanWye, J.L. Roach, J.A. Whitehead, G.J. Wyckoff, K.J. Kolell, and D.L. Crawford. (2004) FunnyBase: a systems level functional annotation of Fundulus ESTs for the analysis of gene expression. *BMC Genomics* **5,** 96.

CAP3 was used to cluster EST sequences with a 30 bp overlap and 75 percent similarity.

1. Pavy, N., C. Paule, L. Parsons, J.A. Crow, M.J. Morency, J. Cooke, J.E. Johnson, E. Noumen, C. Guillet-Claude, Y. Butterfield, S. Barber, G. Yang, J. Liu, J. Stott, R. Kirkpatrick, A. Siddiqui, R. Holt, M. Marra, A. Seguin, E. Retzel, J. Bousquet, and J. MacKay. (2005) Generation, annotation, analysis and database integration of 16,500 white spruce EST clusters. *BMC Genomics* **6,** 144.

The Phrap assembly parameters used were minmatch 50 and minscore 100. Only reads with at least 100 nt of sequence with a quality score above 20 were assembled. Phrap contigs were evaluated for chimeric sequences, and reassembled after removing chimeric reads. Consensus sequences are classified as being of block, staircase, or dumbell shape. Chimeric reads are removed based on their similarity to the consensus sequence and to the individual reads in the contig or if BLAST hits to different proteins are found to be adjacent in the read. Contigs were joined based on mate pairs.

1. Pinto, L.R., K.M. Oliveira, E.C. Ulian, A.A. Garcia, and A.P. de Souza. (2004) Survey in the sugarcane expressed sequence tag database (SUCEST) for simple sequence repeats. *Genome* **47,** 795-804.

Assembled with CAP3.

1. Pirooznia, M., P. Gong, X. Guan, L.S. Inouye, K. Yang, E.J. Perkins, and Y. Deng. (2007) Cloning, analysis and functional annotation of expressed sequence tags from the Earthworm Eisenia fetida. *BMC Bioinformatics* **8 Suppl 7,** S7.

Assembled with Phrap

1. Poustka, A.J., D. Groth, S. Hennig, S. Thamm, A. Cameron, A. Beck, R. Reinhardt, R. Herwig, G. Panopoulou, and H. Lehrach. (2003) Generation, annotation, evolutionary analysis, and database integration of 20,000 unique sea urchin EST clusters. *Genome Res* **13,** 2736-2746.

The sequences were clustered based on BLAST results and assembled with CAP3.

1. Pratt, L.H., C. Liang, M. Shah, F. Sun, H. Wang, S.P. Reid, A.R. Gingle, A.H. Paterson, R. Wing, R. Dean, R. Klein, H.T. Nguyen, H.M. Ma, X. Zhao, D.T. Morishige, J.E. Mullet, and M.M. Cordonnier-Pratt. (2005) Sorghum expressed sequence tags identify signature genes for drought, pathogenesis, and skotomorphogenesis from a milestone set of 16,801 unique transcripts. *Plant Physiol* **139,** 869-884.

Phrap assembly of 3' ESTs where the 5' were added afterwards, i.e. "To reduce the frequency of poorly assembled TU [contig], members of each TU were resubmitted to phrap one TU at a time."

1. Quilang, J., S. Wang, P. Li, J. Abernathy, E. Peatman, Y. Wang, L. Wang, Y. Shi, R. Wallace, X. Guo, and Z. Liu. 2007. Generation and analysis of ESTs from the eastern oyster, Crassostrea virginica Gmelin and identification of microsatellite and SNP markers. *BMC Genomics* **8:** 157.

Vector *NTI Advance*™ 10 (Invitrogen Corporation, 2005), which uses an implementation of CAP3 (www.invitrogen.com)

1. Ralph, S.G., H.J. Chun, N. Kolosova, D. Cooper, C. Oddy, C.E. Ritland, R. Kirkpatrick, R. Moore, S. Barber, R.A. Holt, S.J. Jones, M.A. Marra, C.J. Douglas, K. Ritland, and J. Bohlmann. 2008. A conifer genomics resource of 200,000 spruce (Picea spp.) ESTs and 6,464 high-quality, sequence-finished full-length cDNAs for Sitka spruce (Picea sitchensis). *BMC Genomics* **9:** 484.

A separate CAP3 assembly of the 5' and 3' ESTs.

1. Ramirez, M., Graham, M.A., Blanco-Lopez, L., Silvente, S., Medrano-Soto, A., Blair, M.W., Hernandez, G., Vance, C.P. and Lara, M. (2005) Sequencing and analysis of common bean ESTs. Building a foundation for functional genomics. *Plant Physiol*, **137**, 1211-1227.

Phrap for assembly with mismatch of 50 and minscore of 100

1. Ramsey, J.S., Wilson, A.C., de Vos, M., Sun, Q., Tamborindeguy, C., Winfield, A., Malloch, G., Smith, D.M., Fenton, B., Gray, S.M. *et al.* (2007) Genomic resources for Myzus persicae: EST sequencing, SNP identification, and microarray design. *BMC Genomics*, **8**, 423.

Clustered with TribeMCL and assembled with CAP3

1. Rudd, S. (2005) openSputnik--a database to ESTablish comparative plant genomics using unsaturated sequence collections. *Nucleic Acids Res* **33,** D622-627.

ESTs were clustered with a suffix tree approach and assembled with CAP3.

1. Sczyrba, A., M. Beckstette, A.H. Brivanlou, R. Giegerich, and C.R. Altmann. (2005) XenDB: full length cDNA prediction and cross species mapping in Xenopus laevis. *BMC Genomics* **6,** 123.

Used a suffix array based clustering approach.

1. Shoemaker, R., P. Keim, L. Vodkin, E. Retzel, S.W. Clifton, R. Waterston, D. Smoller, V. Coryell, A. Khanna, J. Erpelding, X. Gai, V. Brendel, C. Raph-Schmidt, E.G. Shoop, C.J. Vielweber, M. Schmatz, D. Pape, Y. Bowers, B. Theising, J. Martin, M. Dante, T. Wylie, and C. Granger. (2002) A compilation of soybean ESTs: generation and analysis. *Genome* **45,** 329-338.

CAP3 and ZmDBAssembler (www.amdb.iastate.edu/smdb/EST/assembly.html)

1. Shi, Y.H., S.W. Zhu, X.Z. Mao, J.X. Feng, Y.M. Qin, L. Zhang, J. Cheng, L.P. Wei, Z.Y. Wang, and Y.X. Zhu. (2006) Transcriptome profiling, molecular biological, and physiological studies reveal a major role for ethylene in cotton fiber cell elongation. *Plant Cell* **18,** 651-664.

Assembled with Stackpack.

1. Sonstegard, T.S., A.V. Capuco, J. White, C.P. Van Tassell, E.E. Connor, J. Cho, R. Sultana, L. Shade, J.E. Wray, K.D. Wells, and J. Quackenbush. (2002) Analysis of bovine mammary gland EST and functional annotation of the Bos taurus gene index. *Mamm Genome* **13,** 373-379.

Assembled with TGICL.

1. Sterck, L., S. Rombauts, S. Jansson, F. Sterky, P. Rouze, and Y. Van de Peer. (2005) EST data suggest that poplar is an ancient polyploid. *New Phytol* **167,** 165-170.

TGICL was used for assembly with the minimal overlap set to 40. The coding frame was determined with FrameD. For each gene family, all members were aligned with each other at the protein level with CLUSTALW. Starting from the cleaned alignments, *K*S was estimated using a maximum-likelihood approach as implemented in the program codeml is part of the paml package.

1. Tanguy, A., Bierne, N., Saavedra, C., Pina, B., Bachere, E., Kube, M., Bazin, E., Bonhomme, F., Boudry, P., Boulo, V. *et al.* (2008) Increasing genomic information in bivalves through new EST collections in four species: development of new genetic markers for environmental studies and genome evolution. *Gene*, **408**, 27-36.

Assembled with TGICL

1. Trail, F., Xu, J.R., San Miguel, P., Halgren, R.G. and Kistler, H.C. (2003) Analysis of expressed sequence tags from Gibberella zeae (anamorph Fusarium graminearum). *Fungal Genet Biol*, **38**, 187-197.

Assembled with Stackpack

1. Udall, J.A., J.M. Swanson, K. Haller, R.A. Rapp, M.E. Sparks, J. Hatfield, Y. Yu, Y. Wu, C. Dowd, A.B. Arpat, B.A. Sickler, T.A. Wilkins, J.Y. Guo, X.Y. Chen, J. Scheffler, E. Taliercio, R. Turley, H. McFadden, P. Payton, N. Klueva, R. Allen, D. Zhang, C. Haigler, C. Wilkerson, J. Suo, S.R. Schulze, M.L. Pierce, M. Essenberg, H. Kim, D.J. Llewellyn, E.S. Dennis, D. Kudrna, R. Wing, A.H. Paterson, C. Soderlund, and J.F. Wendel. (2006) A global assembly of cotton ESTs. *Genome Res* **16,** 441-450.

Assembled with an early version of PAVE.

1. Uenishi, H., T. Eguchi, K. Suzuki, T. Sawazaki, D. Toki, H. Shinkai, N. Okumura, N. Hamasima, and T. Awata. (2004) PEDE (Pig EST Data Explorer): construction of a database for ESTs derived from porcine full-length cDNA libraries. *Nucleic Acids Res* **32,** D484-488.

Assembled with TGICL.

1. Vettore, A.L., F.R. da Silva, E.L. Kemper, G.M. Souza, A.M. da Silva, M.I. Ferro, F. Henrique-Silva, E.A. Giglioti, M.V. Lemos, L.L. Coutinho, et al. 2003. Analysis and functional annotation of an expressed sequence tag collection for tropical crop sugarcane. *Genome Res* **13:** 2725-2735.

Assembled with CAP3

1. Vizcaino, J.A., Gonzalez, F.J., Suarez, M.B., Redondo, J., Heinrich, J., Delgado-Jarana, J., Hermosa, R., Gutierrez, S., Monte, E., Llobell, A. *et al.* (2006) Generation, annotation and analysis of ESTs from Trichoderma harzianum CECT 2413. *BMC Genomics*, **7**, 193.

Assembled with CAP3

1. Vihtelic, T.S., J.M. Fadool, J. Gao, K.A. Thornton, D.R. Hyde, and G. Wistow. 2005. Expressed sequence tag analysis of zebrafish eye tissues for NEIBank. *Mol Vis* **11:** 1083-1100.

Assembled with GRIST, which is a program developed for this project (Winstow et al. 2002. Molecular Vision, 8:164).

1. von Schalburg, K.R., Leong, J., Cooper, G.A., Robb, A., Beetz-Sargent, M.R., Lieph, R., Holt, R.A., Moore, R., Ewart, K.V., Driedzic, W.R. *et al.* (2008) Rainbow Smelt (Osmerus mordax) Genomic Library and EST Resources. *Mar Biotechnol (NY)*.

Two-stage phrap and a CAP (i.e. two assemblies)

1. Wlaschin, K.F., P.M. Nissom, L. Gatti Mde, P.F. Ong, S. Arleen, K.S. Tan, A. Rink, B. Cham, K. Wong, M. Yap, and W.S. Hu. (2005) EST sequencing for gene discovery in Chinese hamster ovary cells. *Biotechnol Bioeng* **91,** 592-606.

Used msi_trim_phred-Phrap, a modified version of Phred/Phrap/Consed, created at the University of Minnesota Supercomputing Institute.

1. Yang, J., Chen, L., Wang, L., Zhang, W., Liu, T. and Jin, Q. (2007) TrED: the Trichophyton rubrum Expression Database. *BMC Genomics*, **8**, 250.

Assembled with TGICL

1. Yu, J.K., Sun, Q., Rota, M.L., Edwards, H., Tefera, H. and Sorrells, M.E. (2006) Expressed sequence tag analysis in tef (Eragrostis tef (Zucc) Trotter). *Genome*, **49**, 365-372.

Assembled with Phrap.

1. Zhang, D., D.W. Choi, S. Wanamaker, R.D. Fenton, A. Chin, M. Malatrasi, Y. Turuspekov, H. Walia, E.D. Akhunov, P. Kianian, et al. (2004) Construction and evaluation of cDNA libraries for large-scale expressed sequence tag sequencing in wheat (Triticum aestivum L.). *Genetics* **168,** 595-608.

Assembled with CAP3, with the HarvEST program. (harvest.ucr.edu)

1. Zhang, H., Sreenivasulu, N., Weschke, W., Stein, N., Rudd, S., Radchuk, V., Potokina, E., Scholz, U., Schweizer, P., Zierold, U. *et al.* (2004) Large-scale analysis of the barley transcriptome based on expressed sequence tags. *Plant J*, **40**, 276-290.

Assembled with StackPack.

### B. Next-generation sequencing of ESTs

1. Bainbridge, M.N., Warren, R.L., Hirst, M., Romanuik, T., Zeng, T., Go, A., Delaney, A., Griffith, M., Hickenbotham, M., Magrini, V. *et al.* 2006. Analysis of the prostate cancer cell line LNCaP transcriptome using a sequencing-by-synthesis approach. *BMC Genomics*, **7**: 246.

181,279 454 ESTs were blasted against the human genome.

1. Barbazuk, W.B., S.J. Emrich, H.D. Chen, L. Li, and P.S. Schnable. 2007. SNP discovery via 454 transcriptome sequencing. *Plant J* **51:** 910-918.

Using a 454, 260k and 280k ESTs were generated from two different maize inbred lines. There was no assembly step. POLYBAYES was used to determine SNPs.

1. Cheung, F., B.J. Haas, S.M. Goldberg, G.D. May, Y. Xiao, and C.D. Town. 2006. Sequencing Medicago truncatula expressed sequenced tags using 454 Life Sciences technology. *BMC Genomics* **7:** 272.

Using 454 GS20 and TGICL, 252k high quality sequences and assembled. The average length was 92 bp. The assembly resulted in 33,865 contigs and 150,734 singletons. The majority of the 184,599 unique sequences were less than 150 bp in length and there were only 2 contigs that had between 51-100 ESTs (none had more). Using information about the adaptors, identified 3' and 5' reads (though these can not be linked).

1. Emrich, S.J., W.B. Barbazuk, L. Li, and P.S. Schnable. 2007. Gene discovery and annotation using LCM-454 transcriptome sequencing. *Genome Res* **17:** 69-73.

261k 454 maize ESTs were generated with an average length of 101 bp. The ESTs were aligned to Sanger ESTs and the MAGIs (assembled genes). A conclusion on p71 is "This substantial 3'-enrichment provides confidence that the number of novel transcripts detected in this study is not substantially overestimated. "

1. Eveland, A.L., D.R. McCarty, and K.E. Koch. 2008. Transcript profiling by 3'-untranslated region sequencing resolves expression of gene families. *Plant Physiol* **146:** 32-44.

Using 454 GS20 and CAP3, 229K 3' ESTs were generated and assembled. The biggest contig was 2500 ESTs. They aligned these to EST contigs in order to annotate them. Up to 10000 ESTs matched to a given transcript.

1. Glazov, E.A., P.A. Cottee, W.C. Barris, R.J. Moore, B.P. Dalrymple, and M.L. Tizard. 2008. A microRNA catalog of the developing chicken embryo identified by a deep sequencing approach. *Genome Res* **18:** 957-964.

Used Solexa reads to investigate microRNA.

1. Monaghan, J.R., Epp, L.G., Putta, S., Page, R.B., Walker, J.A., Beachy, C.K., Zhu, W., Pao, G.M., Verma, I.M., Hunter, T. *et al.* (2009) Microarray and cDNA sequence analysis of transcription during nerve-dependent limb regeneration. *BMC Biol*, **7**, 1.

454 FLX generated the ESTs for two treatments resulting in 90k and 230k sequences of average size 215 bp. PACE was used to cluster and CAP3 used to assemble.

1. Novaes, E., Drost, D.R., Farmerie, W.G., Pappas, G.J., Jr., Grattapaglia, D., Sederoff, R.R. and Kirst, M. (2008) High-throughput gene and SNP discovery in Eucalyptus grandis, an uncharacterized genome. *BMC Genomics*, **9**, 312.

Two GS-20 and one GS-FLX 454 runs. Assembled with a combination of Newbler and Paracel Transcript Assembler. They compare the output of the GS-20, GS-FLX and Sanger based on length of contigs and number of reads in contigs.

1. Ohtsu, K., Smith, M.B., Emrich, S.J., Borsuk, L.A., Zhou, R., Chen, T., Zhang, X., Timmermans, M.C., Beck, J., Buckner, B. *et al.* (2007) Global gene expression analysis of the shoot apical meristem of maize (Zea mays L.). *Plant J*, **52**, 391-404.

454 ESTs and blasted against a retrotransposon library.

1. Rothberg, J.M. and J.H. Leamon. 2008. The development and impact of 454 sequencing. *Nat Biotechnol* **26:** 1117-1124.

454 sequencing (Roche, Basel) is described and compared with Illumina's Genome Analyzer and ABI's SOLID. The Illumina's Genome Analyzer was developed by Solexa (Cambridge, UK and "brought to market" by Illumina. SOLID is technology by Church, Shendure et al (Shendure, et al. 2005. Science 309: 1728) and "brought to market" by ABI.

1. Shin, H., M. Hirst, M.N. Bainbridge, V. Magrini, E. Mardis, D.G. Moerman, M.A. Marra, D.L. Baillie, and S.J. Jones. 2008. Transcriptome analysis for Caenorhabditis elegans based on novel expressed sequence tags. *BMC Biol* **6:** 30.

Using 454, 300k sequences were generated with an average 100 bp length. There was no assembly step as they aligned the sequences to the transcripts and genome sequence.

1. Torres, T.T., M. Metta, B. Ottenwalder, and C. Schlotterer. 2008. Gene expression profiling by massively parallel sequencing. *Genome Res* **18:** 172-177.

GS-20 454 ESTs were mapped to the Drosophila melanogaster sequenced genome. They experimented with 3' reads from digested and nebulized samples. They found that longer reads map better to the genome; that ESTs shorter than ~80 bp or longer than 300 bp were under-represented; that it is highly reproducible and nebulization does not cause no strong bias.

1. Vera, J.C., C.W. Wheat, H.W. Fescemyer, M.J. Frilander, D.L. Crawford, I. Hanski, and J.H. Marden. 2008. Rapid transcriptome characterization for a nonmodel organism using 454 pyrosequencing. *Mol Ecol* **17:** 1636-1647.

Using 454 reads and Seqman Pro for assembly, generated 608k ESTs with average length of 110 bp. It assembled into 48,354 contigs and 59,943 singletons. The average depth of the longest 4800 contigs was 6.5 with length of 348-2849 bp. They could not use the Newbler assembler due to proprietary primer sequences (see their supplemental information). The Seqman Pro will not assemble more then 34,000 sequences at a time, so the assembly was done in stages. However, the latest Seqman software will assemble it.

1. Weber, A.P., Weber, K.L., Carr, K., Wilkerson, C. and Ohlrogge, J.B. (2007) Sampling the Arabidopsis transcriptome with massively parallel pyrosequencing. *Plant Physiol*, **144**, 32-42.

Used 454 GS20 to produce 541,852 ESTs. They experimented using Newbler, StackPack and CAP3 to assemble the sequences, though had problems with the big contigs.

## 5. Related papers

### A. Assorted

*This section contains related papers, not necessarily specific to ESTs, e.g. Zhang et al 2005 describes software for detecting SNPs in PCR products. The papers are a mix of software, analysis and surveys. Emphasize is on papers related to plants.*

1. Al-Shahrour, F., R. Diaz-Uriarte, and J. Dopazo (2004) FatiGO: a web tool for finding significant associations of Gene Ontology terms with groups of genes. *Bioinformatics* **20,** 578-580.
2. Altshuler, D., V.J. Pollara, C.R. Cowles, W.J. Van Etten, J. Baldwin, L. Linton, and E.S. Lander. (2000) An SNP map of the human genome generated by reduced representation shotgun sequencing. *Nature* **407,** 513-516.

Defined NQS (neighborhood quality standard) to be a phred score >= 20 and the 5 bases on both sides to have phred scores >= 15. True SNPs in humans is 1 in 1300bp.

1. Barzuza, T., Beckmann, J.S., Shamir, R. and Pe'er, I. (2005) Typing without calling the allele: a strategy for inferring SNP haplotypes. *Eur J Hum Genet*.
2. Beaudoing, E. and D. Gautheret (2001) Identification of alternate polyadenylation sites and analysis of their tissue distribution using EST data. *Genome Res* **11,** 1520-1526.
3. Bouck, A. and Vision, T. (2007) The molecular ecologist's guide to expressed sequence tags. *Mol Ecol*, **16**, 907-924.
4. Brockman, J.M., P. Singh, D. Liu, S. Quinlan, J. Salisbury, and J.H. Graber. (2005) **PACdb**: PolyA Cleavage Site and 3'-UTR Database. *Bioinformatics* **21,** 3691-3693.
5. Cannon, S.B. and Young, N.D. (2003) **OrthoParaMap**: distinguishing orthologs from paralogs by integrating comparative genome data and gene phylogenies. *BMC Bioinformatics*, **4**, 35.
6. Cerutti, H. (2003) RNA interference: traveling in the cell and gaining functions? *Trends Genet*, **19**, 39-46.
7. Chen, Z., W. Wang, X.B. Ling, J.J. Liu, and L. Chen. (2006) **GO-Diff**: mining functional differentiation between EST-based transcriptomes. *BMC Bioinformatics* **7,** 72.
8. Chen, Y.A., Lin, C.C., Wang, C.D., Wu, H.B. and Hwang, P.I. (2007) An optimized procedure greatly improves EST vector contamination removal. *BMC Genomics*, **8**, 416.
9. Chou, A. and J. Burke. (1999) **CRAWview**: for viewing splicing variation, gene families, and polymorphism in clusters of ESTs and full-length sequences. *Bioinformatics* **15,** 376-381.
10. Cuff, J.A., Birney, E., Clamp, M.E. and Barton, G.J. (2000) **ProtEST**: protein multiple sequence alignments from expressed sequence tags. *Bioinformatics*, **16**, 111-116.

ProtEST takes as input a query sequence, compares it against EMBL-EST and creates a multiple alignment with the query, EST contigs and protein matches. It uses various BLAST programs, Phrap, ESTWISE (Birney, unpublished), SCANPS (Barton, 1993, CABIOS 9, 729), and CLUSTALW. (barton.ebi.ac.uk/servers/protest.html)

1. Del Val, C., K.H. Glatting, and S. Suhai. (2003) **cDNA2Genome**: a tool for mapping and annotating cDNAs. *BMC Bioinformatics* **4,** 39.
2. Enright, A.J. and Ouzounis, C.A. (2000) **GeneRAGE**: a robust algorithm for sequence clustering and domain detection. *Bioinformatics*, **16**, 451-457.
3. Friedel, C.C., Jahn, K.H., Sommer, S., Rudd, S., Mewes, H.W. and Tetko, I.V. (2005) Support vector machines for separation of mixed plant-pathogen EST collections based on codon usage. *Bioinformatics*, **21**, 1383-1388.
4. Fukunishi, Y. and Y. Hayashizaki. (2001) Amino acid translation program for full-length cDNA sequences with frameshift errors. *Physiol Genomics* **5,** 81-87.
5. Ge, B., S. Gurd, T. Gaudin, C. Dore, P. Lepage, E. Harmsen, T.J. Hudson, and T. Pastinen. (2005) Survey of allelic expression using EST mining. *Genome Res* **15,** 1584-1591.
6. Gemund, C., C. Ramu, B. Altenberg-Greulich, and T.J. Gibson. (2001) **Gene2EST**: a BLAST2 server for searching expressed sequence tag (EST) databases with eukaryotic gene-sized queries. *Nucleic Acids Res* **29,** 1272-1277.
7. Green, R.E., B.P. Lewis, R.T. Hillman, M. Blanchette, L.F. Lareau, A.T. Garnett, D.C. Rio, and S.E. Brenner. (2003) Widespread predicted nonsense-mediated mRNA decay of alternatively-spliced transcripts of human normal and disease genes. *Bioinformatics* **19 Suppl 1,** i118-121.
8. Hayes, B.J., Nilsen, K., Berg, P.R., Grindflek, E. and Lien, S. (2007) SNP detection exploiting multiple sources of redundancy in large EST collections improves validation rates. *Bioinformatics*.
9. Huang, Y., J. Pumphrey, and A.R. Gingle. (2005) **ESTminer**: a Web interface for mining EST contig and cluster databases. *Bioinformatics* **21,** 669-670.
10. Iacono, M., F. Mignone, and G. Pesole. (2005) uAUG and uORFs in human and rodent 5'untranslated mRNAs. *Gene* **349,** 97-105.
11. Irizarry, K., V. Kustanovich, C. Li, N. Brown, S. Nelson, W. Wong, and C.J. Lee. (2000) Genome-wide analysis of single-nucleotide polymorphisms in human expressed sequences. *Nat Genet* **26,** 233-236.
12. Jongeneel, C.V. (2000) Searching the expressed sequence tag (EST) databases: panning for genes. *Brief Bioinform* **1,** 76-92.
13. Katayama, S., Tomaru, Y., Kasukawa, T., Waki, K., Nakanishi, M., Nakamura, M., Nishida, H., Yap, C.C., Suzuki, M., Kawai, J. *et al.* (2005) Antisense transcription in the mammalian transcriptome. *Science*, **309**, 1564-1566.
14. Koski, L.B., M.W. Gray, B.F. Lang, and G. Burger. (2005) **AutoFACT**: an automatic functional annotation and classification tool. *BMC Bioinformatics* **6,** 151.
15. Larsson, T.P., Murray, C.G., Hill, T., Fredriksson, R. and Schioth, H.B. (2005) Comparison of the current RefSeq, Ensembl and EST databases for counting genes and gene discovery. *FEBS Lett*, **579**, 690-698.
16. Lavorgna, G., L. Sessa, A. Guffanti, L. Lassandro, and G. Casari. (2004) **AntiHunter**: searching BLAST output for EST antisense transcripts. *Bioinformatics* **20,** 583-585.
17. Liu, D. and J.H. Graber. (2006) Quantitative comparison of EST libraries requires compensation for systematic biases in cDNA generation. *BMC Bioinformatics* **7,** 77.
18. Lottaz, C., C. Iseli, C.V. Jongeneel, and P. Bucher. (2003) Modeling sequencing errors by combining Hidden Markov models. *Bioinformatics* **19 Suppl 2,** II103-II112
19. Malde, K. and Jonassen, I. (2008) Repeats and EST analysis for new organisms. *BMC Genomics*, **9**, 23.

They investigate the effect of repeat masking on assembly, and conclude that it should be limited to specifies specific libraries and eliminated entirely.

1. Maquat, L.E. (2005) Nonsense-mediated mRNA decay in mammals. *J Cell Sci*, **118**, 1773-1776.
2. Mignone, F., Gissi, C., Liuni, S. and Pesole, G. (2002) Untranslated regions of mRNAs. *Genome Biol*, **3**, REVIEWS0004.
3. Murray, C.G., T.P. Larsson, T. Hill, R. Bjorklind, R. Fredriksson, and H.B. Schioth. (2005) Evaluation of EST-data using the genome assembly. *Biochem Biophys Res Commun* **331,** 1566-1576.
4. Nadershahi, A., S.C. Fahrenkrug, and L.B. Ellis. (2004) Comparison of computational methods for identifying translation initiation sites in EST data. *BMC Bioinformatics* **5,** 14.
5. Nilsson, R.H., B. Rajashekar, K.H. Larsson, and B.M. Ursing. (2004) **galaxieEST**: addressing EST identity through automated phylogenetic analysis. *BMC Bioinformatics* **5,** 87.
6. Pesole, G., S. Liuni, G. Grillo, F. Licciulli, F. Mignone, C. Gissi, and C. Saccone. (2002) **UTRdb** and UTRsite: specialized databases of sequences and functional elements of 5' and 3' untranslated regions of eukaryotic mRNAs. Update 2002. *Nucleic Acids Res* **30,** 335-340.
7. Quackenbush, J., J. Cho, D. Lee, F. Liang, I. Holt, S. Karamycheva, B. Parvizi, G. Pertea, R. Sultana, and J. White. (2001) The TIGR Gene Indices: analysis of gene transcript sequences in highly sampled eukaryotic species. *Nucleic Acids Res* **29,** 159-164.
8. Nagaraj, S.H., Gasser, R.B. and Ranganathan, S. (2007) A hitchhiker's guide to expressed sequence tag (EST) analysis. *Brief Bioinform*, **8**, 6-21.
9. Neeman, Y., Dahary, D., Levanon, E.Y., Sorek, R. and Eisenberg, E. (2005) Is there any sense in antisense editing? *Trends Genet*, **21**, 544-547.
10. Peregrin-Alvarez, J.M., Yam, A., Sivakumar, G. and Parkinson, J. (2005) PartiGeneDB--collating partial genomes. *Nucleic Acids Res*, **33**, D303-307.

PartiGene is pipeline software (see Parkinson et al., Section II).

1. Rafalski, A. (2002) Applications of single nucleotide polymorphisms in crop genetics. *Curr Opin Plant Biol* **5,** 94-100.
2. Remm, M., Storm, C.E. and Sonnhammer, E.L. (2001) Automatic clustering of orthologs and in-paralogs from pairwise species comparisons. *J Mol Biol*, **314**, 1041-1052.
3. Romualdi, C., Bortoluzzi, S., D'Alessi, F. and Danieli, G.A. (2003) **IDEG6**: a web tool for detection of differentially expressed genes in multiple tag sampling experiments. *Physiol Genomics*, **12**, 159-162.
4. Sanderson, M.J. and McMahon, M.M. (2007) Inferring angiosperm phylogeny from EST data with widespread gene duplication. *BMC Evol Biol*, **7** Suppl 1, S3.
5. Schneeberger, K., K. Malde, E. Coward, and I. Jonassen. (2005) Masking repeats while clustering ESTs. *Nucleic Acids Res* **33,** 2176-2180.
6. Schlueter, J.A., P. Dixon, C. Granger, D. Grant, L. Clark, J.J. Doyle, and R.C. Shoemaker. (2004) Mining EST databases to resolve evolutionary events in major crop species. *Genome* **47,** 868-876.
7. Shafer, P., D.M. Lin, and G. Yona. (2006) **EST2Prot**: Mapping EST sequences to proteins. *BMC Genomics* **7,** 41.
8. Shah, S.C. and A. Kusiak. (2004) Data mining and genetic algorithm based gene/SNP selection. *Artif Intell Med* **31,** 183-196.
9. Sherry, S.T., M.H. Ward, M. Kholodov, J. Baker, L. Phan, E.M. Smigielski, and K. Sirotkin. (2001) **dbSNP**: the NCBI database of genetic variation. *Nucleic Acids Res* **29,** 308-311.
10. Sonnhammer, E.L. and Koonin, E.V. (2002) Orthology, paralogy and proposed classification for paralog subtypes. *Trends Genet*, **18**, 619-620.
11. Sorek, R. and H.M. Safer. (2003) A novel algorithm for computational identification of contaminated EST libraries. *Nucleic Acids Res* **31,** 1067-1074.
12. Stekel, D.J., Git, Y. and Falciani, F. (2000) The comparison of gene expression from multiple cDNA libraries. *Genome Res*, **10**, 2055-2061.
13. Susko, E. and A.J. Roger. (2004) Estimating and comparing the rates of gene discovery and expressed sequence tag (EST) frequencies in EST surveys. *Bioinformatics* **20,** 2279-2287.
14. Wang, J.P., B.G. Lindsay, L. Cui, P.K. Wall, J. Marion, J. Zhang, and C.W. dePamphilis. (2005) Gene capture prediction and overlap estimation in EST sequencing from one or multiple libraries. *BMC Bioinformatics* **6,** 300.
15. Wang, J.P., B.G. Lindsay, J. Leebens-Mack, L. Cui, K. Wall, W.C. Miller, and C.W. dePamphilis. (2004) EST clustering error evaluation and correction. *Bioinformatics* **20,** 2973-2984.
16. Wu, X., M.G. Walker, J. Luo, and L. Wei. (2005) **GBA** server: EST-based digital gene expression profiling. *Nucleic Acids Res* **33,** W673-676.
17. Wu, J., Mao, X., Cai, T., Luo, J. and Wei, L. (2006) **KOBAS** server: a web-based platform for automated annotation and pathway identification. *Nucleic Acids Res*, **34**, W720-724.
18. Yang, H.H., Y. Hu, M. Edmonson, K. Buetow, and M.P. Lee. (2003) Computation method to identify differential allelic gene expression and novel imprinted genes. *Bioinformatics* **19,** 952-955.
19. Zhang, B.H., X.P. Pan, Q.L. Wang, G.P. Cobb, and T.A. Anderson. (2005) Identification and characterization of new plant microRNAs using EST analysis. *Cell Res* **15,** 336-360.
20. Zmasek, C.M. and Eddy, S.R. (2001) A simple algorithm to infer gene duplication and speciation events on a gene tree. *Bioinformatics*, **17**, 821-828.

### B. Alternative Splicing software

1. Boue, S., Letunic, I. and Bork, P. (2003) Alternative splicing and evolution. *Bioessays*, **25**, 1031-1034.
2. Brendel, V., Xing, L. and Zhu, W. (2004) Gene structure prediction from consensus spliced alignment of multiple ESTs matching the same genomic locus. *Bioinformatics*, **20**, 1157-1169.
3. Cartegni, L., Chew, S.L. and Krainer, A.R. (2002) Listening to silence and understanding nonsense: exonic mutations that affect splicing. *Nat Rev Genet*, **3**, 285-298.
4. Cartegni, L., Wang, J., Zhu, Z., Zhang, M.Q. and Krainer, A.R. (2003) **ESEfinder**: A web resource to identify exonic splicing enhancers. *Nucleic Acids Res*, **31**, 3568-3571.
5. Cusack, B.P. and Wolfe, K.H. (2005) Changes in alternative splicing of human and mouse genes are accompanied by faster evolution of constitutive exons. *Mol Biol Evol*, **22**, 2198-2208.
6. Dralyuk, I., Brudno, M., Gelfand, M.S., Zorn, M. and Dubchak, I. (2000) **ASDB**: database of alternatively spliced genes. *Nucleic Acids Res*, **28**, 296-297.
7. Enerly, E., Sheng, Z. and Li, K.B. (2005) Natural antisense as potential regulator of alternative initiation, splicing and termination. *In Silico Biol*, **5**, 367-377.
8. Eyras, E., M. Caccamo, V. Curwen, and M. Clamp. 2004. **ESTGenes**: alternative splicing from ESTs in Ensembl. *Genome Res* **14:** 976-987.
9. Gupta, S., D. Zink, B. Korn, M. Vingron, and S.A. Haas. (2004) Genome wide identification and classification of alternative splicing based on EST data. *Bioinformatics* **20,** 2579-2585.
10. Huang, H.D., J.T. Horng, F.M. Lin, Y.C. Chang, and C.C. Huang. (2005) **SpliceInfo**: an information repository for mRNA alternative splicing in human genome. *Nucleic Acids Res* **33,** D80-85.
11. Huang, H.D., Horng, J.T., Lee, C.C. and Liu, B.J. (2003) **ProSplicer**: a database of putative alternative splicing information derived from protein, mRNA and expressed sequence tag sequence data. *Genome Biol*, **4**, R29.
12. Huang, Y.H., Chen, Y.T., Lai, J.J., Yang, S.T. and Yang, U.C. (2002) **PALS db**: Putative Alternative Splicing database. *Nucleic Acids Res*, **30**, 186-190.
13. Itoh, H., T. Washio, and M. Tomita. (2004) Computational comparative analyses of alternative splicing regulation using full-length cDNA of various eukaryotes. *Rna* **10,** 1005-1018.
14. Kan, Z., D. States, and W. Gish. (2002) Selecting for functional alternative splices in ESTs. *Genome Res* **12,** 1837-1845.
15. Kent, W.J. and Zahler, A.M. (2000) The **intronerator**: exploring introns and alternative splicing in Caenorhabditis elegans. *Nucleic Acids Res*, **28**, 91-93.
16. Kim, N., S. Shin, and S. Lee. (2005) **ECgene**: genome-based EST clustering and gene modeling for alternative splicing. *Genome Res* **15,** 566-576.
17. Kim, N., S. Shin, and S. Lee. (2004) **ASmodeler**: gene modeling of alternative splicing from genomic alignment of mRNA, EST and protein sequences. *Nucleic Acids Res* **32,** W181-186.
18. Krause, A., S.A. Haas, E. Coward, and M. Vingron. 2002. **SYSTERS,** GeneNest, SpliceNest: exploring sequence space from genome to protein. *Nucleic Acids Res* **30:** 299-300.
19. Lareau, L.F., Green, R.E., Bhatnagar, R.S. and Brenner, S.E. (2004) The evolving roles of alternative splicing. *Curr Opin Struct Biol*, **14**, 273-282.
20. Lee, C., L. Atanelov, B. Modrek, and Y. Xing. (2003) **ASAP:** the Alternative Splicing Annotation Project. *Nucleic Acids Res* **31,** 101-105.
21. Lewis, B.P., Green, R.E. and Brenner, S.E. (2003) Evidence for the widespread coupling of alternative splicing and nonsense-mediated mRNA decay in humans. *Proc Natl Acad Sci U S A*, **100**, 189-192.
22. Leipzig, J., P. Pevzner, and S. Heber. (2004) The Alternative Splicing Gallery (**ASG**): bridging the gap between genome and transcriptome. *Nucleic Acids Res* **32,** 3977-3983.
23. Modrek, B. and C. Lee. (2002) A genomic view of alternative splicing. *Nat Genet* **30,** 13-19.
24. Pospisil, H., A. Herrmann, R.H. Bortfeldt, and J.G. Reich. (2004) **EASED:** Extended Alternatively Spliced EST Database. *Nucleic Acids Res* **32,** D70-74.
25. Resch, A., Xing, Y., Alekseyenko, A., Modrek, B. and Lee, C. (2004) Evidence for a subpopulation of conserved alternative splicing events under selection pressure for protein reading frame preservation. *Nucleic Acids Res*, **32**, 1261-1269.
26. Resch, A., Xing, Y., Modrek, B., Gorlick, M., Riley, R. and Lee, C. (2004) Assessing the impact of alternative splicing on domain interactions in the human proteome. *J Proteome Res*, **3**, 76-83.
27. Stamm, S., Ben-Ari, S., Rafalska, I., Tang, Y., Zhang, Z., Toiber, D., Thanaraj, T.A. and Soreq, H. (2005) Function of alternative splicing. *Gene*, **344**, 1-20.
28. Thanaraj, T.A., S. Stamm, F. Clark, J.J. Riethoven, V. Le Texier, and J. Muilu. (2004) ASD: the Alternative Splicing Database. *Nucleic Acids Res* **32,** D64-69.
29. Yeo, G.W., Van Nostrand, E., Holste, D., Poggio, T. and Burge, C.B. (2005) Identification and analysis of alternative splicing events conserved in human and mouse. *Proc Natl Acad Sci U S A*, **102**, 2850-2855.

### C. Full Length cDNA

1. Gerhard, D.S., Wagner, L., Feingold, E.A., Shenmen, C.M., Grouse, L.H., Schuler, G., Klein, S.L., Old, S., Rasooly, R., Good, P. *et al.* (2004) The status, quality, and expansion of the NIH full-length cDNA project: the Mammalian Gene Collection (MGC). *Genome Res*, **14**, 2121-2127.
2. Iida, K., Seki, M., Sakurai, T., Satou, M., Akiyama, K., Toyoda, T., Konagaya, A. and Shinozaki, K. (2004) Genome-wide analysis of alternative pre-mRNA splicing in Arabidopsis thaliana based on full-length cDNA sequences. *Nucleic Acids Res*, **32**, 5096-5103.
3. Kawai, J., Shinagawa, A., Shibata, K., Yoshino, M., Itoh, M., Ishii, Y., Arakawa, T., Hara, A., Fukunishi, Y., Konno, H. *et al.* (2001) Functional annotation of a full-length mouse cDNA collection. *Nature*, **409**, 685-690.
4. Kikuchi, S., Satoh, K., Nagata, T., Kawagashira, N., Doi, K., Kishimoto, N., Yazaki, J., Ishikawa, M., Yamada, H., Ooka, H. *et al.* (2003) Collection, mapping, and annotation of over 28,000 cDNA clones from japonica rice. *Science*, **301**, 376-379.
5. Morin, R.D., Chang, E., Petrescu, A., Liao, N., Griffith, M., Kirkpatrick, R., Butterfield, Y.S., Young, A.C., Stott, J., Barber, S. *et al.* (2006) Sequencing and analysis of 10,967 full-length cDNA clones from Xenopus laevis and Xenopus tropicalis reveals post-tetraploidization transcriptome remodeling. *Genome Res*.
6. Osato, N., Itoh, M., Konno, H., Kondo, S., Shibata, K., Carninci, P., Shiraki, T., Shinagawa, A., Arakawa, T., Kikuchi, S. *et al.* (2002) A computer-based method of selecting clones for a full-length cDNA project: simultaneous collection of negligibly redundant and variant cDNAs. *Genome Res*, **12**, 1127-1134.
7. Osato, N., Yamada, H., Satoh, K., Ooka, H., Yamamoto, M., Suzuki, K., Kawai, J., Carninci, P., Ohtomo, Y., Murakami, K. *et al.* (2003) Antisense transcripts with rice full-length cDNAs. *Genome Biol*, **5**, R5.
8. Seki, M., Narusaka, M., Kamiya, A., Ishida, J., Satou, M., Sakurai, T., Nakajima, M., Enju, A., Akiyama, K., Oono, Y. *et al.* (2002) Functional annotation of a full-length Arabidopsis cDNA collection. *Science*, **296**, 141-145.
9. Stapleton, M., Carlson, J., Brokstein, P., Yu, C., Champe, M., George, R., Guarin, H., Kronmiller, B., Pacleb, J., Park, S. *et al.* (2002) A Drosophila full-length cDNA resource. *Genome Biol*, **3**, RESEARCH0080.

## 6. Typical references in EST papers.

*These are non-EST papers used for annotation of ESTs, i.e. not listed in previous sections but are referred to in many of the descriptions.*

1. Altschul, S.F., T.L. Madden, A.A. Schaffer, J. Zhang, Z. Zhang, W. Miller, and D.J. Lipman. (1997) Gapped **BLAST** and PSI-BLAST: a new generation of protein database search programs. *Nucleic Acids Res* **25,** 3389-3402.
2. Ashburner, M., Ball, C.A., Blake, J.A., Botstein, D., Butler, H., Cherry, J.M., Davis, A.P., Dolinski, K., Dwight, S.S., Eppig, J.T. *et al.* (2000) **Gene ontology**: tool for the unification of biology. The Gene Ontology Consortium. *Nat Genet*, **25**, 25-29.
3. Bairoch, A., R. Apweiler, C.H. Wu, W.C. Barker, B. Boeckmann, S. Ferro, E. Gasteiger, H. Huang, R. Lopez, M. Magrane, M.J. Martin, D.A. Natale, C. O'Donovan, N. Redaschi, and L.S. Yeh. (2005) The Universal Protein Resource (**UniProt**). *Nucleic Acids Res* **33,** D154-159.
4. Benson, D.A., Karsch-Mizrachi, I., Lipman, D.J., Ostell, J. and Wheeler, D.L. (2005) **GenBank**. *Nucleic Acids Res*, **33**, D34-38.
5. Boguski, M.S., T.M. Lowe, and C.M. Tolstoshev. (1993) **dbEST**--database for "expressed sequence tags". *Nat Genet* **4,** 332-333.
6. **CAT** from DoubleTwist (www.doubletwist.com/).
7. Camon, E., M. Magrane, D. Barrell, V. Lee, E. Dimmer, J. Maslen, D. Binns, N. Harte, R. Lopez, and R. Apweiler. (2004) The Gene Ontology Annotation (**GOA**) Database: sharing knowledge in Uniprot with Gene Ontology. *Nucleic Acids Res* 32 Database issue: D262-266.
8. Conesa, A., Gotz, S., Garcia-Gomez, J.M., Terol, J., Talon, M. and Robles, M. (2005) **Blast2GO**: a universal tool for annotation, visualization and analysis in functional genomics research. *Bioinformatics*, **21**, 3674-3676.
9. **cross_match** (boseman.mbt.washington.edu/phrap.docs/phrap.html)
10. Kent, W.J. (2002) **BLAT**--the BLAST-like alignment tool. *Genome Res*, **12**, 656-664.
11. Pearson, W.R. (1994) Using the **FASTA** program to search protein and DNA sequence databases. *Methods Mol Biol*, **24**, 307-331.
12. Goldman, N. and Z. Yang. (1994) A codon-based model of nucleotide substitution for protein-coding DNA sequences. *Mol Biol Evol* **11,** 725-736.
13. Gordon, D., C. Abajian, and P. Green. (1998) **Consed**: a graphical tool for sequence finishing. *Genome Res* **8,** 195-202.
14. Maglott, D., Ostell, J., Pruitt, K.D. and Tatusova, T. (2005) **Entrez** Gene: gene-centered information at NCBI. *Nucleic Acids Res*, **33**, D54-58.
15. Mulder, N.J., R. Apweiler, T.K. Attwood, A. Bairoch, A. Bateman, D. Binns, P. Bradley, P. Bork, P. Bucher, L. Cerutti, et. al. 2005. **InterPro**, progress and status in 2005. *Nucleic Acids Res* **33:** D201-205.
16. Ning, Z., Cox, A.J. and Mullikin, J.C. (2001) **SSAHA**: a fast search method for large DNA databases. *Genome Res*, **11**, 1725-1729.
17. **NCBI Vector** database (ftp://ftp.ncbi.nih.gov/blast/db/FASTA/vector.gz). Vector masking/VECTOR/ UniVec (www.ncbi.nlm.nih.gov/VecScreen/VecScreen.html)
18. Pontius, J., L. Wagner, G. Schuler. (2003) **UniGene**: a unified view of the transcriptome. In: The NCBI Handbook. Bethesda (MD): National Center for Biotechnology Information.
19. Ewing, B., L. Hillier, M.C. Wendl, and P. Green. (1998) Base-calling of automated sequencer traces using **phred**. I. Accuracy assessment. *Genome Res* **8,** 175-185.
20. Jurka, J. (2000) **Repbase** update: a database and an electronic journal of repetitive elements. *Trends Genet*, **16**, 418-420. (www.girinst.org)
21. **RepeatMasker** (www.repeatmasker.org)
22. **Seqclean** (www.tigr.org/tdb/tgi/software) for vector trimming.
23. **Seqman (**www.dnastar.com/products/seqmanpro.php)
24. Staden, R. (1996) The **Staden** sequence analysis package. *Mol Biotechnol*, **5**, 233-241. (staden.sourceforge.net)
25. Stekel, D.J., Git, Y. and Falciani, F. (2000) The comparison of gene expression from multiple cDNA libraries. *Genome Res*, **10**, 2055-2061.
26. Thompson, J.D., D.G. Higgins, and T.J. Gibson. (1994) **CLUSTAL W**: improving the sensitivity of progressive multiple sequence alignment through sequence weighting, position-specific gap penalties and weight matrix choice. *Nucleic Acids Res* **22,** 4673-4680.
27. **vmatch** (www.vmatch.de)
28. Yang, Z. 1997. **PAML**: a program package for phylogenetic analysis by maximum likelihood. *Comput Appl Biosci* **13:** 555-556.
29. Zhang, Z., S. Schwartz, L. Wagner, and W. Miller. (2000) A greedy algorithm for aligning DNA sequences. *J Comput Biol* **7,** 203-214. **(MegaBLAST,** mpiblast.lanl.gov)
