# Supplementary material for: PAVE: Program for assembling and viewing ESTs
Source: BMC Genomics. 2009 Aug 26;10:400. doi: 10.1186/1471-2164-10-400 (PMC2748094; doi:10.1186/1471-2164-10-400)
Supplement: Additional file 2 — Parameters and log files. The parameters and content the of log files for the maize, trichomes and benchmark assemblies. [file 1471-2164-10-400-S2.doc]

# Additional File 2: PAVE parameters

This supplemental contains the PAVE parameters and log files for the assemblies from the paper.

Though we execute PAVE on 64-bit machines, we found that using the standard CAP3 works better than the 64-bit CAP3.

### Maize Sanger EST assembly:

The following parameters varies from the defaults:

CPUs 4

SELF_JOIN 50 96 30

CLIQUE 150 99 20

TC1 150 98 20

TC2 150 96 40

TC3 120 94 40

TC4 120 94 40

BLAST_ARGS -e1e-25 -W32 -D3 -FF -v100

CAP_ARGS -p 85 -y 70 -b 80 -o 49 -t 10000 > /dev/null 2>&1

The following is the log file of the execution :

User-provided EST self-BLAST in /agcol/not_backed_up/blasts/ZmGB_est_selfblast.tab

Converting /agcol/not_backed_up/blasts/ZmGB_est_selfblast.tab to /agcol/not_backed_up/blasts/ZmGB_est_selfblast.tab.conv started Mon Feb 9 11:44:06 2009

Completed Mon Feb 9 11:59:28 2009

>>>Initial self-BLAST HV Sort Started Mon Feb 9 11:59:30 2009

Initial self-BLAST HV Sort Completed Mon Feb 9 12:35:15 2009

>>>Cliques started Mon Feb 9 12:35:15 2009

Computing BLAST containment started Mon Feb 9 12:35:15 2009

21775 ESTs buried.

Completed Mon Feb 9 14:04:22 2009

Parsing sorted self-BLAST started Mon Feb 9 14:04:24 2009

108417190 self-BLAST HSPs processed

11765968 EST pairs accepted 32863208 EST pairs failed

Completed Mon Feb 9 15:55:52 2009

Finding mate cliques Mon Feb 9 15:55:52 2009

81194 clones (vertices) 717155 clone pairs (edges)

81194 vertices, 81194 edges

40332 cliques found

Creating cliques table Mon Feb 9 15:58:55 2009

49840 overlapping mate-pairs are to be made into contigs

Completed Mon Feb 9 15:59:06 2009

Assembling cliques Mon Feb 9 15:59:06 2009

36269 assemblies attempted 36208 successful

35313 one 898 two 0 >two 59 none contigs created

Completed Mon Feb 9 21:43:36 2009

Finding loner cliques Mon Feb 9 21:43:37 2009

389789 vertices, 389789 edges

836886 cliques found

Creating cliques table Mon Feb 9 22:06:13 2009

Completed Mon Feb 9 22:08:16 2009

Assembling cliques Mon Feb 9 22:08:16 2009

26331 assemblies attempted 26331 successful

61644 one 898 two 0 >two 59 none contigs created

Completed Tue Feb 10 06:50:10 2009

Creating initial contigs Tue Feb 10 06:50:14 2009

470241 initial contigs (i.e. assembled cliques+mate-pairs)

Completed Tue Feb 10 07:06:57 2009

>>>Transitive closure 1 started Tue Feb 10 07:06:58 2009

BLAST Started Tue Feb 10 07:07:07 2009

Blasting 337413 old contigs 470241 new contigs

Completed Tue Feb 10 07:34:35 2009

Finding transitive closures Tue Feb 10 07:35:09 2009

1986694 pairs accepted, approximately 7604985 rejected.

330666 CSSs (vertices) 1986694 pairs (edges)

29673 transitive closures

Completed Tue Feb 10 08:59:15 2009

Assembling transitive closures Tue Feb 10 11:26:57 2009

668 clone(s) with mate-pairs assembling in same direction

360117 assemblies attempted 272725 successful 43818 new contigs

Completed Sat Feb 14 16:45:22 2009

>>>Transitive closure 2 started Sat Feb 14 16:45:22 2009

BLAST Started Sat Feb 14 16:45:25 2009

Blasting 83408 old contigs 43818 new contigs

Completed Sat Feb 14 16:52:36 2009

Finding transitive closures Sat Feb 14 16:53:36 2009

60726 pairs accepted, approximately 438091 rejected.

59794 CSSs (vertices) 60726 pairs (edges)

16306 transitive closures

Completed Sat Feb 14 16:57:10 2009

Assembling transitive closures Sat Feb 14 17:25:53 2009

692 clone(s) with mate-pairs assembling in same direction

53637 assemblies attempted 23851 successful 15828 new contigs

Completed Sun Feb 15 08:12:56 2009

>>>Transitive closure 3 started Sun Feb 15 08:12:56 2009

BLAST Started Sun Feb 15 08:12:59 2009

Blasting 87547 old contigs 15828 new contigs

Completed Sun Feb 15 08:15:02 2009

Finding transitive closures Sun Feb 15 08:15:12 2009

38403 pairs accepted, approximately 308842 rejected.

41975 CSSs (vertices) 38403 pairs (edges)

12310 transitive closures

Completed Sun Feb 15 08:17:33 2009

Assembling transitive closures Sun Feb 15 08:40:00 2009

704 clone(s) with mate-pairs assembling in same direction

37256 assemblies attempted 10401 successful 8141 new contigs

Completed Sun Feb 15 20:19:35 2009

>>>Transitive closure 4 started Sun Feb 15 20:19:35 2009

BLAST Started Sun Feb 15 20:19:37 2009

Blasting 84833 old contigs 8141 new contigs

Completed Sun Feb 15 20:20:49 2009

Finding transitive closures Sun Feb 15 20:21:11 2009

20540 pairs accepted, approximately 265489 rejected.

24576 CSSs (vertices) 20540 pairs (edges)

8135 transitive closures

Completed Sun Feb 15 20:22:31 2009

Assembling transitive closures Sun Feb 15 20:36:03 2009

705 clone(s) with mate-pairs assembling in same direction

4841 merge short contained contigs

19949 assemblies attempted 4992 successful 4397 new contigs

Completed Mon Feb 16 01:48:13 2009

>>>Finalize contigs Mon Feb 16 01:48:17 2009

Contigs with multiple clones: 45213 joined by 50 n's: 10806

Contigs with one mate-pair: 5986 joined by 50 n's: 4140

Contig sizes: =2 3-5 6-10 11-20 21-50 51-100 101-1k >1k

13488 13501 8492 6514 6125 2090 986 3

Contigs: 51199

Singletons: 36783

Total: 87982

797619 ESTs in assembly, with 105574 buried.

Finished PAVE assembly ZmGB on localhost Mon Feb 16 06:19:07 2009

>>>Total PAVE time 6:18:40:58 (day:hr:min:sec)

### Trichomes 454 EST assembly:

In order to prevent the assembly of large contigs, the assembly goes through multiple clustering stages in order to slowly reduce the TC parameters; in other words, ESTs can be continually buried in smaller contigs and then merged, in order to prevent big contigs from being assembled. The parameters are:

CPUs 4

CAP_BURY_EC_THRESHOLD 200

BLAST_BURY_MISMATCH 1

LARGE_THRESHOLD 450000

CLIQUE 95 97 20

TC1 93 96 20

TC2 90 94 30

TC3 85 92 30

TC4 85 87 30

TC5 83 85 30

TC6 80 85 50

TC7 70 85 50

TC8 70 85 50

BLAST_ARGS -e1e-25 -W28 -FF -v100

CAP_ARGS -p 80 -y 70 -b 80 -f 8 -o 49 -t 10000 > /dev/null

The log file:

ESTs written to ests.fasta from the following libraries:

415559 from library arcPw

EST library totals:

415559 ESTs

0 mate-pairs

0 unmated 3' & 5' ESTs

415559 ESTs of unknown direction

User-provided EST self-BLAST in /agcol/not_backed_up/blasts/tri_arc.selfblast.tab

>>>Cliques started Sun Feb 15 11:03:43 2009

Computing BLAST containment started Sun Feb 15 11:03:43 2009

179474 ESTs buried.

Completed Sun Feb 15 19:48:18 2009

Parsing sorted self-BLAST started Sun Feb 15 19:48:21 2009

514644157 self-BLAST HSPs processed

41766063 EST pairs accepted 18840259 EST pairs failed

Completed Mon Feb 16 03:39:41 2009

No mate pairs exist, will not look for mate cliques.

Finding loner cliques Mon Feb 16 03:39:42 2009

289903 vertices, 289903 edges

2265908 cliques found

Creating cliques table Wed Feb 18 07:13:06 2009

Completed Wed Feb 18 07:29:32 2009

Assembling cliques Wed Feb 18 07:29:32 2009

15634 assemblies attempted 15634 successful

15634 one 0 two 0 >two 0 none contigs created

Completed Thu Feb 19 05:14:33 2009

Creating initial contigs Thu Feb 19 05:14:44 2009

157567 initial contigs (i.e. assembled cliques+mate-pairs)

Completed Thu Feb 19 06:42:15 2009

>>>Transitive closure 1 started Thu Feb 19 06:42:15 2009

BLAST Started Thu Feb 19 06:42:17 2009

Blasting 141933 old contigs 157567 new contigs

Completed Thu Feb 19 06:56:03 2009

Finding transitive closures Thu Feb 19 06:56:06 2009

238477 pairs accepted, approximately 293736 rejected.

98363 CSSs (vertices) 238477 pairs (edges)

21233 transitive closures

Completed Thu Feb 19 06:56:40 2009

Assembling transitive closures Thu Feb 19 07:03:01 2009

80190 assemblies attempted 74057 successful 23082 new contigs

Completed Fri Feb 20 12:48:55 2009

>>>Transitive closure 2 started Fri Feb 20 12:48:55 2009

BLAST Started Fri Feb 20 12:48:57 2009

Blasting 60428 old contigs 23082 new contigs

Completed Fri Feb 20 12:51:25 2009

Finding transitive closures Fri Feb 20 12:51:27 2009

4617 pairs accepted, approximately 31526 rejected.

7025 CSSs (vertices) 4617 pairs (edges)

2708 transitive closures

Completed Fri Feb 20 12:51:29 2009

Assembling transitive closures Fri Feb 20 12:52:40 2009

4417 assemblies attempted 3700 successful 2715 new contigs

Completed Fri Feb 20 16:20:45 2009

>>>Transitive closure 3 started Fri Feb 20 16:20:45 2009

BLAST Started Fri Feb 20 16:20:48 2009

Blasting 77095 old contigs 2715 new contigs

Completed Fri Feb 20 16:21:08 2009

Finding transitive closures Fri Feb 20 16:21:09 2009

3524 pairs accepted, approximately 23573 rejected.

5042 CSSs (vertices) 3524 pairs (edges)

1878 transitive closures

Completed Fri Feb 20 16:21:11 2009

Assembling transitive closures Fri Feb 20 16:22:06 2009

3389 assemblies attempted 2371 successful 1791 new contigs

Completed Fri Feb 20 18:47:09 2009

>>>Transitive closure 4 started Fri Feb 20 18:47:09 2009

BLAST Started Fri Feb 20 18:47:11 2009

Blasting 75648 old contigs 1791 new contigs

Completed Fri Feb 20 18:47:25 2009

Finding transitive closures Fri Feb 20 18:47:26 2009

2660 pairs accepted, approximately 19737 rejected.

3117 CSSs (vertices) 2660 pairs (edges)

988 transitive closures

Completed Fri Feb 20 18:47:28 2009

Assembling transitive closures Fri Feb 20 18:48:09 2009

2480 assemblies attempted 1162 successful 689 new contigs

Completed Fri Feb 20 20:10:10 2009

>>>Transitive closure 5 started Fri Feb 20 20:10:10 2009

BLAST Started Fri Feb 20 20:10:12 2009

Blasting 75588 old contigs 689 new contigs

Completed Fri Feb 20 20:10:23 2009

Finding transitive closures Fri Feb 20 20:10:25 2009

1900 pairs accepted, approximately 18358 rejected.

2916 CSSs (vertices) 1900 pairs (edges)

1139 transitive closures

Completed Fri Feb 20 20:10:27 2009

Assembling transitive closures Fri Feb 20 20:10:53 2009

1891 assemblies attempted 727 successful 664 new contigs

Completed Fri Feb 20 21:38:13 2009

>>>Transitive closure 6 started Fri Feb 20 21:38:13 2009

BLAST Started Fri Feb 20 21:38:15 2009

Blasting 74886 old contigs 664 new contigs

Completed Fri Feb 20 21:38:26 2009

Finding transitive closures Fri Feb 20 21:38:27 2009

4186 pairs accepted, approximately 16032 rejected.

6160 CSSs (vertices) 4186 pairs (edges)

2428 transitive closures

Completed Fri Feb 20 21:38:29 2009

Assembling transitive closures Fri Feb 20 21:38:56 2009

4114 assemblies attempted 2251 successful 2013 new contigs

Completed Sat Feb 21 00:34:51 2009

>>>Transitive closure 7 started Sat Feb 21 00:34:51 2009

BLAST Started Sat Feb 21 00:34:53 2009

Blasting 71286 old contigs 2013 new contigs

Completed Sat Feb 21 00:35:05 2009

Finding transitive closures Sat Feb 21 00:35:06 2009

4786 pairs accepted, approximately 12757 rejected.

7032 CSSs (vertices) 4786 pairs (edges)

2755 transitive closures

Completed Sat Feb 21 00:35:08 2009

Assembling transitive closures Sat Feb 21 00:35:45 2009

4571 assemblies attempted 2657 successful 2331 new contigs

Completed Sat Feb 21 03:56:48 2009

>>>Transitive closure 8 started Sat Feb 21 03:56:48 2009

BLAST Started Sat Feb 21 03:56:50 2009

Blasting 68311 old contigs 2331 new contigs

Completed Sat Feb 21 03:57:03 2009

Finding transitive closures Sat Feb 21 03:57:04 2009

1361 pairs accepted, approximately 11962 rejected.

2063 CSSs (vertices) 1361 pairs (edges)

799 transitive closures

Completed Sat Feb 21 03:57:06 2009

Assembling transitive closures Sat Feb 21 03:57:49 2009

1361 assemblies attempted 43 successful 37 new contigs

Completed Sat Feb 21 04:54:40 2009

>>>Finalize contigs Sat Feb 21 04:54:50 2009

Contig sizes: =2 3-5 6-10 11-20 21-50 51-100 101-1k >1k

9925 8249 2860 1463 1049 445 412 33

Contigs: 24436

Singletons: 46163

Total: 70599

415559 ESTs in assembly, with 145325 buried.

Finished PAVE assembly tri_arc2 on localhost Sat Feb 21 11:57:22 2009

>>>Total PAVE time 6:00:54:29 (day:hr:min:sec)

### Benchmark Sanger assembly:

The parameters are:

CPUs 4

SELF_JOIN 50 94 20

CLIQUE 150 99 20

TC1 150 98 20

TC2 150 95 30

BLAST_ARGS -e1e-20 -W32 -D3 -FF -G1 -E2

CAP_ARGS -p 90 -y 70 -b 80 -o 49 -t 10000 > /dev/null 2>&1

The log file is:

>>>Initial self-BLAST Started Thu Feb 19 14:24:51 2009

Self-BLAST output file: /opt/users/matthew/pave_dev/projects/Bmk1/Bmk1_ests.selfblast

Initial self-BLAST Completed Thu Feb 19 15:33:47 2009

>>>Initial self-BLAST HV Sort Started Thu Feb 19 15:33:47 2009

Initial self-BLAST HV Sort Completed Thu Feb 19 15:34:39 2009

>>>Cliques started Thu Feb 19 15:34:39 2009

Computing BLAST containment started Thu Feb 19 15:34:39 2009

0 ESTs buried.

Completed Thu Feb 19 15:37:01 2009

Parsing sorted self-BLAST started Thu Feb 19 15:37:01 2009

1940467 self-BLAST HSPs processed

700795 EST pairs accepted 258095 EST pairs failed

Completed Thu Feb 19 15:39:23 2009

Finding mate cliques Thu Feb 19 15:39:23 2009

24257 clones (vertices) 249842 clone pairs (edges)

24257 vertices, 24257 edges

14057 cliques found

Creating cliques table Thu Feb 19 15:40:16 2009

5870 overlapping mate-pairs are to be made into contigs

Completed Thu Feb 19 15:40:20 2009

Assembling cliques Thu Feb 19 15:40:20 2009

4814 assemblies attempted 4769 successful

Completed Thu Feb 19 16:14:49 2009

Finding loner cliques Thu Feb 19 16:14:49 2009

0 vertices, 0 edges

0 cliques found

Creating cliques table Thu Feb 19 16:14:52 2009

Completed Thu Feb 19 16:14:52 2009

Assembling cliques Thu Feb 19 16:14:52 2009

0 assemblies attempted 0 successful

Completed Thu Feb 19 16:14:52 2009

Creating initial contigs Thu Feb 19 16:14:53 2009

39566 initial contigs (i.e. assembled cliques+mate-pairs)

Completed Thu Feb 19 16:15:22 2009

>>>Transitive closure 1 started Thu Feb 19 16:15:22 2009

BLAST Started Thu Feb 19 16:15:23 2009

Blasting 17400 old contigs 39566 new contigs

Completed Thu Feb 19 16:16:10 2009

Finding transitive closures Thu Feb 19 16:16:11 2009

70836 pairs accepted, approximately 40952 rejected.

21460 CSSs (vertices) 70836 pairs (edges)

4763 transitive closures

Completed Thu Feb 19 16:16:19 2009

Assembling transitive closures Thu Feb 19 16:16:35 2009

16909 assemblies attempted 16253 successful 4927 new contigs

Completed Thu Feb 19 17:34:52 2009

>>>Transitive closure 2 started Thu Feb 19 17:34:52 2009

BLAST Started Thu Feb 19 17:34:53 2009

Blasting 986 old contigs 4927 new contigs

Completed Thu Feb 19 17:35:15 2009

Finding transitive closures Thu Feb 19 17:35:15 2009

518 pairs accepted, approximately 587 rejected.

899 CSSs (vertices) 518 pairs (edges)

424 transitive closures

Completed Thu Feb 19 17:35:15 2009

Assembling transitive closures Thu Feb 19 17:35:32 2009

285 merge short contained contigs

502 assemblies attempted 312 successful 307 new contigs

Completed Thu Feb 19 17:38:44 2009

>>>Finalize contigs Thu Feb 19 17:38:45 2009

Contigs with multiple clones: 4957 joined by 50 n's: 3461

Contigs with one mate-pair: 644 joined by 50 n's: 53

Contig sizes: =2 3-5 6-10 11-20 21-50 51-100 101-1k >1k

644 1537 1966 869 468 89 28 0

Contigs: 5601

Singletons: 0

Total: 5601

61706 ESTs in assembly, with 4186 buried.

Finished PAVE assembly Bmk1 on taq Thu Feb 19 17:49:38 20

>>>Total PAVE time 0:03:25:06 (day:hr:min:sec)
