# Supplementary material for: PAVE: Program for assembling and viewing ESTs
Source: BMC Genomics. 2009 Aug 26;10:400. doi: 10.1186/1471-2164-10-400 (PMC2748094; doi:10.1186/1471-2164-10-400)
Supplement: Additional file 3 — Benchmark contigs. A description of the steps and parameters used to create the benchmark contigs. [file 1471-2164-10-400-S3.doc]

# Additional file 3: Benchmark dataset

To compare the assemblies, a set of benchmark contigs were created that contained ESTs that align to a subset of the KOME rice FL-cDNAs [1]. The ESTs consisted of 583,053 KOME ESTs and an additional 68,920 rice ESTs[2] for which we have quality files. The FL-cDNAs were compared to each other with MegaBlast [3], and when two ESTs aligned for over 200 bases at 95% identity, the shorter EST was removed. The 651,973 ESTs were filtered to retain only those with a mate-pair and at least 400 bases in length.

The filtered EST set was compared with the filtered FL-cDNAs using MegaBlast, and then the output was filtered to create the benchmark set of ESTs as follows: (i) each EST aligned to a FL-cDNA with at least 97% similarity over 350 bases, less than 10 mismatches, and less than 20 bases overhang, (ii) both mates had to align to the same FL-cDNA in opposite directions, (iii) each EST had to overlap at least one other EST in the set of ESTs for the given FL-cDNA. This resulted in 61,706 ESTs aligning to 5437 FL-cDNAs, i.e. 5437 benchmark contigs.

The CAP3[4] assembly parameters "-p 90 -y 70 -b 80 -o 49 -t 10000" were used for all three assemblies.

# References

1. Kikuchi S, Satoh K, Nagata T, Kawagashira N, Doi K, Kishimoto N, Yazaki J, Ishikawa M, Yamada H, Ooka H *et al*: **Collection, mapping, and annotation of over 28,000 cDNA clones from japonica rice**. *Science* 2003, **301**(5631):376-379.

2. Jantasuriyarat C, Gowda M, Haller K, Hatfield J, Lu G, Stahlberg E, Zhou B, Li H, Kim H, Yu Y *et al*: **Large-scale identification of expressed sequence tags involved in rice and rice blast fungus interaction**. *Plant Physiol* 2005, **138**(1):105-115.

3. Zhang Z, Schwartz S, Wagner L, Miller W: **A greedy algorithm for aligning DNA sequences**. *J Comput Biol* 2000, **7**(1-2):203-214.

4. Huang X, Madan A: **CAP3: A DNA sequence assembly program**. *Genome Res* 1999, **9**(9):868-877.
